# Short‐Term Risk of Cardiovascular Events in People Newly Diagnosed With Gout

**DOI:** 10.1002/art.42986

**Published:** 2024-10-17

**Authors:** Edoardo Cipolletta, Georgina Nakafero, Pascal Richette, Anthony J. Avery, Mamas A. Mamas, Laila J. Tata, Abhishek Abhishek

**Affiliations:** ^1^ University of Nottingham, Nottingham, United Kingdom, and Polytechnic University of Marche Ancona Italy; ^2^ University of Nottingham Nottingham United Kingdom; ^3^ Hospital Lariboisière, INSERM U1132 Paris France; ^4^ Keele University Keele United Kingdom

## Abstract

**Objective:**

To investigate the temporal association between the first diagnosis of gout and cardiovascular events in the short term.

**Methods:**

We performed a self‐controlled case series analysis and a cohort study using data from linked primary care, hospitalization, and mortality records from the United Kingdom's Clinical Practice Research Database‐GOLD. We included individuals with a new diagnosis of gout either in the primary care or secondary care between January 1, 1997 and December 31, 2020. The first consultation at which gout was diagnosed was the exposure of interest. The main outcome consisted of cardiovascular events (ie, a composite of fatal and nonfatal myocardial infarction, ischemic or hemorrhagic stroke, and transient ischemic attack).

**Results:**

The 4,398 patients (66.9% male, mean age 74.6 years) had a cardiovascular event within at least two years of their first recorded diagnosis of gout. The incidence of cardiovascular events was significantly higher in the 30 days after the first diagnosis of gout compared to baseline (adjusted incidence rate ratio 1.55, 95% confidence interval [CI] 1.33–1.83). Among 76,440 patients (72.9% male, mean age 63.2 years) included in the cohort study, the incidence of cardiovascular events in the 30 days after the first gout diagnosis (31.2 events per 1,000 person‐years, 95% CI 27.1–35.9) was significantly higher than in days 31 to 730 after gout diagnosis (21.6 events per 1,000 person‐years, 95% CI 20.8–22.4) with a rate difference of −9.6 events per 1,000 person‐years (95% CI −14.0 to −5.1).

**Conclusion:**

Individuals had a short‐term increased risk of cardiovascular events in the 30 days following the first consultation at which gout was diagnosed.

## INTRODUCTION

Gout affects 1.0% to 4.0% of adults in Western countries.^1^ Traditionally viewed as a self‐limiting form of arthritis, it is associated with cardiovascular comorbidities.^2^ Hyperuricemia was long considered a potential reason for the excess cardiovascular burden in people with gout, but Mendelian randomization studies and the ALL‐HEART study did not provide evidence about the causal role of hyperuricemia in the pathogenesis of cardiovascular comorbidities.^3^, ^4^ Thus, the potential mechanism linking gout and cardiovascular events may be related to systemic and vascular inflammation during gout flares.

Gout flares have been associated with a transient increase in the risk of cardiovascular events in two separate studies, one from the United Kingdom and another from Australia.^5.6^ However, these studies either included people with long‐standing gout^5^ or people hospitalized for gout flare.^6^ Their findings might be affected by survivor bias, potentially affecting the magnitude of the association. Additionally, there may be potential confounding effects from the regular prescription of nonsteroidal anti‐inflammatory drugs (NSAIDs) or colchicine to either prevent or treat gout flares. This is an important issue because regular NSAID prescription is associated with an increased risk of cardiovascular events, whereas regular colchicine prescriptions may prevent cardiovascular events.^7^, ^8^ Therefore, it is important to assess whether there is a transient increase in cardiovascular events following gout flares, occurring only in the initial period following gout diagnosis, at a time when there is no effect of survivor bias and minimal effect from long‐term anti‐inflammatory drug prescription. The presence of such an association would strongly support early proactive cardiovascular risk management in people newly diagnosed with gout. Currently, there is a discrepancy between international recommendations regarding the screening and the management of cardiovascular risk factors in those newly diagnosed with gout.^9^, ^10^, ^11^, ^12^ The British Society of Rheumatology^12^ and the EULAR^10^ guidelines suggest screening cardiovascular risk factors and cardiovascular comorbidities in all patients with gout and managing them appropriately. Conversely, the American College of Rheumatology^9^ and the American College of Physicians^11^ guidelines do not make any mention of that.

The main aim of this study was to assess whether there is a short‐term increased risk of cardiovascular events in patients diagnosed with newly diagnosed gout. We also estimated the incidence of cardiovascular events in the first two years following the first gout diagnosis to provide absolute incidence rates. In this study, we used the same data source as our previously published study, but the data were restricted to the first recorded consultation for gout.^5^

## PATIENTS AND METHODS

### Study design and study period

A self‐controlled case series (SCCS) study evaluated the temporal association between the first diagnosis of gout (ie, typically a flare consultation at which gout could be diagnosed in primary care or secondary care) and cardiovascular events. In the SCCS study, eligible patients (ie, those with an exposure and an outcome) act as their own controls. Indeed, comparisons are made within individuals and not between them,^13^ and fixed, time‐invariant confounding is accounted for. A cohort study provided estimates of the incidence of cardiovascular events following the first consultation in which gout was diagnosed. The study period spanned from January 1, 1997 to December 31, 2020.

### Data source

Linked primary care, hospitalization, and mortality data were extracted from the Clinical Practice Research Datalink (CPRD)‐GOLD, Hospital Episode Statistics (HES), and Office for National Statistics (ONS) databases, respectively.^14^ CPRD‐GOLD contains anonymized patient data on sociodemographic and lifestyle factors, prescriptions, diagnoses, and test results originating during routine care from more than 18 million individuals in England.^14^

### Source population

Patients were newly diagnosed with gout at 18 years or older who contributed research‐quality data to CPRD‐GOLD, with their primary care records linked to HES and ONS datasets. To minimize the risk of including prevalent gout cases as incident,^15^ we included patients with a new diagnosis of gout at least two years after their registration at the current practice, without prior prescriptions of urate‐lowering therapy in primary care or prior hospitalization for gout in which gout was not the primary reason for hospitalization. Patients whose first primary care record of gout indicated prevalent gout (eg, history of gout, gout monitoring, etc) were also excluded.

### Exposures

We used two definitions to identify the first recorded diagnosis of gout:

- Definition 1 (broad): first primary care consultation or hospitalization with gout as the first discharge diagnosis. Flares are by far the most common presentation of gout,^1^ and therefore, any first record of a new diagnosis of gout can be reasonably considered as a proxy for the first gout flare, which required contact with the health care system.
- Definition 2 (strict): first primary consultation for gout with either NSAID, colchicine, or glucocorticoid prescription on the same date, a first primary care consultation for gout indicating gout flare care, or hospitalization with gout as the first discharge diagnosis.^5^, ^16^, ^17^, ^18^

The broad definition allowed us to capture all consultations at which gout was first diagnosed, whereas the strict definition was used to assess the validity of the findings.^19^, ^20^, ^21^

### Outcomes

The primary outcome was the first cardiovascular event after the study entry defined as acute myocardial infarction, stroke (ischemic or hemorrhagic), or transient ischemic attack. Secondary outcomes assessed separately were acute myocardial infarction, cerebrovascular events (ie, stroke [ischemic or hemorrhagic] or transient ischemic attack), and any cardiovascular event requiring hospitalization or leading to death. Events were ascertained using primary care, hospitalization, and mortality records, as linkage across all data sources has been shown to improve outcome ascertainment.^22^, ^23^, ^24^ The date of the cardiovascular event was the earliest of the above dates.

### Study population

To measure the incidence of cardiovascular events following the first gout diagnosis, two separate cohorts were formed using definitions 1 and 2, respectively. Patients were followed up from their first gout diagnosis to the earliest date of last data collection from the practice, transfer out of practice, end of the study period, up to two years after the first gout diagnosis, cardiovascular event, or death.

**Figure 1:**
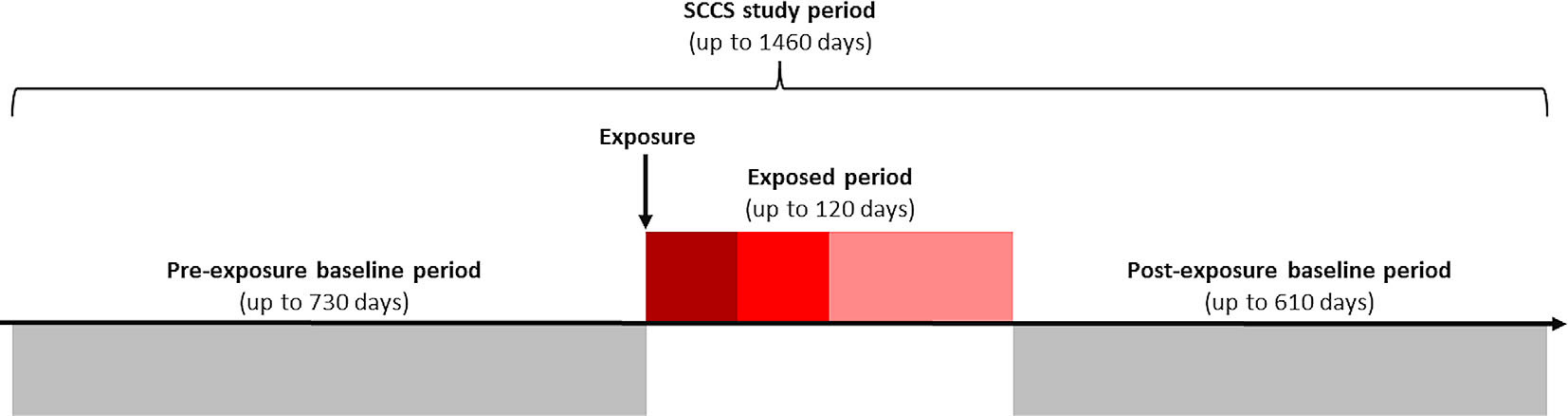
Schematic representation of the SCCS study design. The exposed period (in shades of red) was defined as the period following the exposure. It started on the date of the first gout diagnosis. It ended at the earliest of 120 days after the first gout diagnosis, study end, transfer out, date of last data collection from the general practice, a subsequent gout flare, or death. The baseline period (in gray) consisted of pre‐exposure and post‐exposure periods. The pre‐exposure baseline period (up to 730 days) started at 730 days before the first gout diagnosis. It ended on the day before the first gout diagnosis. The postexposure baseline period (up to 610 days) started on the day after the end of the exposed period. It ended at the earliest date of study end, transfer out, date of last data collection from the general practice, a subsequent gout flare, death, or 610 days after the end of the exposed period. SCCS, self‐controlled case series.

For the SCCS analyses, only patients who experienced a cardiovascular event in the patients’ available registration time up to two years before or up to two years after the first gout diagnosis were included. Two separate SCCS study datasets were formed using exposure definitions 1 and 2, respectively. The exposed period extended up to 120 days after the first gout diagnosis. The baseline period extended up to 730 days before the first gout diagnosis and 615 days after the end of the exposed period (Figure 1). The SCCS study period was censored at the earliest date of study end, transfer out, date of last data collection from the general practice, a subsequent gout flare, or death. Because gout flares typically last for less than two weeks, we considered subsequent gout flares to be present if there was a primary care consultation for gout accompanied by an anti‐inflammatory drug prescription on the same date, primary care consultation for gout at which a Read code for gout flare was recorded, or hospitalization with gout as the primary diagnosis that occurred more than 14 days after the first gout diagnosis.^5^, ^18^

### Statistical analysis

The incidence (with 95% confidence interval [CI]) of cardiovascular events per 1,000 person‐years was calculated on days 1 to 30, 31 to 120, 121 to 730, and 31 to 730 following the first gout diagnosis. Risk differences were calculated for each stratum using days 1 to 30 as the reference category.

In the SCCS analysis, incidence rates and rate difference per 1,000 person‐days and adjusted incidence rate ratios (aIRRs) with 95% CIs were calculated for each stratum of the exposed period (0–30, 31–60, and 61–120 days) compared with the baseline.^13^ We adjusted the SCCS for seasons to account for the seasonal trend of gout flares and cardiovascular events,^25^, ^26^ and age (one‐year bands) to account for the increasing incidence of gout and cardiovascular events with age.^20^ Sample size estimation is presented in Supplementary Material S1.

The SCCS assumptions could be violated by outcomes that increase mortality rates in the short term or when the outcome of interest influences the likelihood of exposure.^27^, ^28^ To evaluate if event‐dependent exposure could be an issue for the SCCS analyses, we plotted the number of outcomes by time before or after gout diagnosis using both exposure definitions. We did not observe a decrease in the occurrence of outcomes in the 30 days immediately before gout diagnosis (Supplementary Material S2). Therefore, an induction period (ie, a period preceding the exposure that was considered separate from the baseline period to minimize potential confounding) was not added to the main analysis.

To assess if event‐dependent observation periods could be an issue for the analyses, we plotted a histogram of the time from the outcome to the actual end of observation in patients who were censored and uncensored using both exposure definitions. A spike close to zero is apparent in the censored data histograms (Supplementary Material S2). This finding indicates the presence of event‐dependent observation periods (censoring on the date of death because of outcome), which we tested further with sensitivity analyses. We performed the following sensitivity analyses:

- used the planned end of observation (eg, 730 days after the first consultation for gout) as the actual end of observation for each case who died of the cardiovascular event and for those who were censored on the date of the next flare^28^;
- excluded patients with fatal cardiovascular events^27^;
- divided the exposure period into shorter intervals;
- included an induction period immediately before the exposure period;
- reduced the baseline period to up to 365 days before and 365 days after the end of the 120‐day exposed period to reduce the potential impact of time‐varying confounders;
- excluded patients with a cardiovascular event on the same date as the first gout diagnosis;
- excluded people with a prescription for NSAIDs, colchicine, or glucocorticoids in the 60 days before first consultation for gout;
- and included only patients with latest serum urate levels >480 μmol/L to increase the confidence in gout diagnosis. To be considered, serum urate must have been measured within five years of the first recorded gout consultation (ie, gout diagnosis). Serum urate measured on the same date as the first recorded gout diagnosis was included because gout flare only reduces serum urate,^29^ and if the serum urate was >480 μmol/L if the measurement was taken on the date of gout flare, it would have been even higher if tested after gout flare resolution.^29^

We also performed stratified analyses according to age (≤70 years old vs >70 years old), sex, prescriptions for anti‐inflammatory drugs (ie, colchicine, glucocorticoids, and NSAIDs) on the date of the first gout diagnosis or within a week after the first gout diagnosis, and hospitalization for gout on the same date as the first gout diagnosis. We evaluated the presence of interactions in these subgroups. We also conducted stratified analyses according to comorbidities (ie, chronic kidney disease, heart failure, diabetes mellitus, and hypertension) and diuretic prescription (ie, below the median and above the median number of prescriptions) to account for the potential confounding effect of these variables in the association between gout diagnosis and cardiovascular events.

To detect unmeasured or unmeasurable sources of bias (eg, the health care–seeking behavior bias), we conducted a negative control analysis^30^ with the first consultation for cataract as the outcome. Negative controls in epidemiologic studies are similar to negative controls in laboratory experiments or a placebo treatment group in a randomized controlled trial.^30^ If an association is observed between the exposure and the negative control outcome that is impossible by the hypothesized mechanism, this suggests that the study is biased.^30^

First‐ever consultation for cataract was our negative control outcome because there is no plausible biologic relationship between gout and the development of cataract. Patients newly diagnosed with gout were included in the SCCS analysis if they also consulted for cataract the for the first time in the 730 days before or after the first gout diagnosis. Similarly, patients diagnosed with gout for the first time were included in the cohort study if they also consulted for cataract for the first time in the 730 days after the first gout diagnosis.

### Ethics, reporting, and patient and public involvement

This study was approved by CPRD Research Data Governance (protocol 20_000233). This manuscript followed the STrengthening the Reporting of OBservational studies in Epidemiology (STROBE) guidelines.^31^ Code lists are provided in Supplementary Material S3. Patient organizations (the UK Gout Society) will be involved in the dissemination plans.

### Data availability

This study used data from the CPRD. These data were provided under a license that does not permit data sharing with third parties. They can be obtained from CPRD.

## RESULTS

### Temporal association between first recorded gout diagnosis and cardiovascular events in the cohort studies

**Figure 2:**
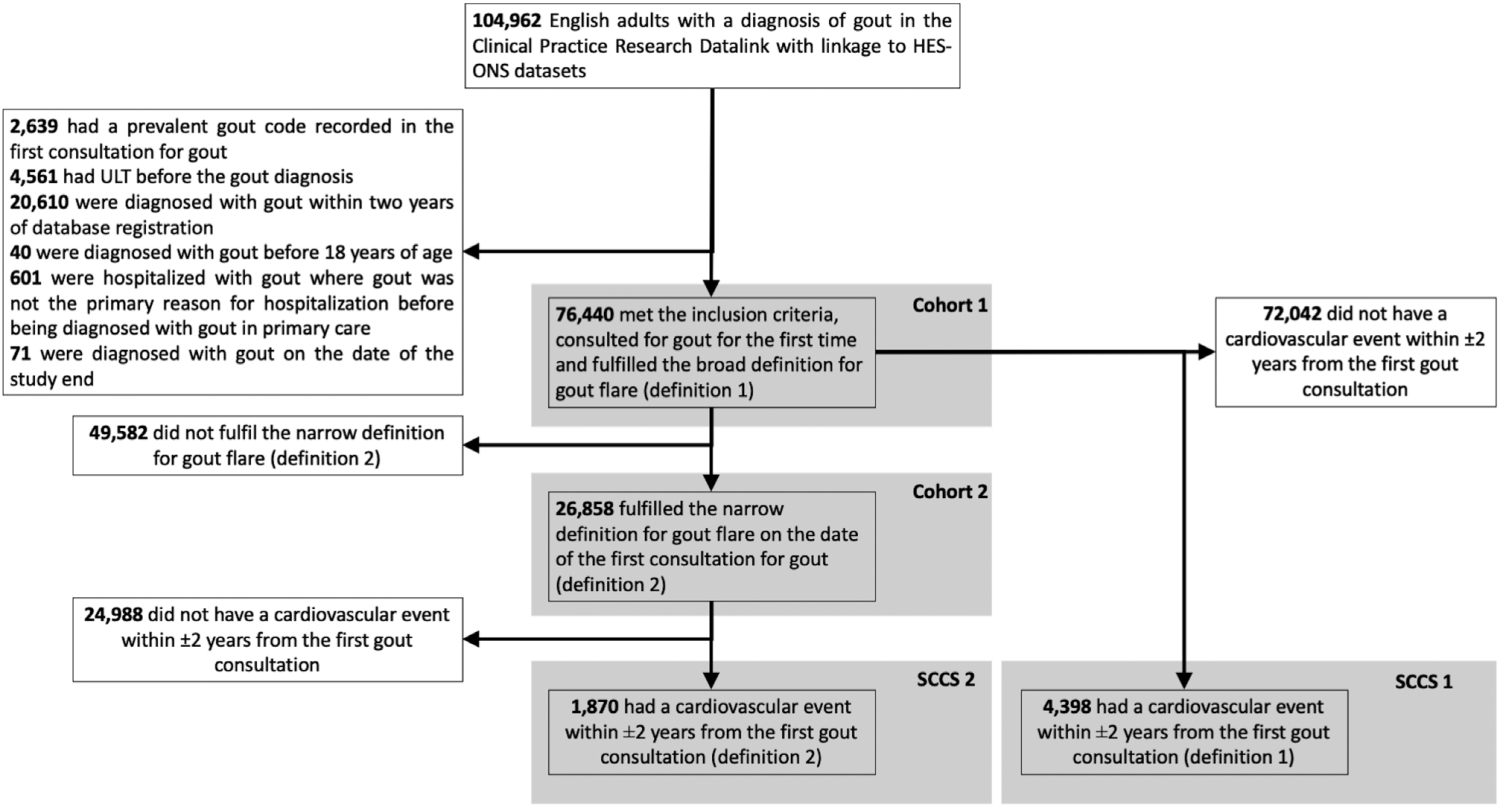
Flow diagram for patient selection. HES, Hospital Episode Statistics; ONS, Office for National Statistics; SCCS, self‐controlled case series; ULT, urate‐lowering therapy.

The 76,440 patients newly diagnosed with gout were included in cohort 1 (Figure 2, Table 1). Among them, 2,956 (3.9%) experienced at least one cardiovascular event within the next two years. The incidence of first cardiovascular event, myocardial infarction, and cerebrovascular events was 22.1 (95% CI 21.3–22.9), 9.4 (95% CI 8.9–9.9), and 13.0 (95% CI 12.4–13.6) per 1,000 person‐years, respectively (Table 2). There were 1,123 (4.2%) cardiovascular events in two years after gout diagnosis among the 26,858 patients newly diagnosed with gout, meeting the strict definition. The incidence of cardiovascular events was comparable with those in cohort 1 (Table 2).

**Table 1:** Baseline clinical and demographic data of patients with gout included in the study*

Patient data	Cohort 1^a^ (n = 76,440)	Cohort 2^b^ (n = 26,858)	SCCS 1^c^ (n = 4,398)	SCCS 2^d^ (n = 1,870)
Age, mean (SD), y	63.2 (15.4)	64.8 (15.2)	74.6 (11.5)	75.2 (11.0)
Female, n (%)	20,674 (27.1)	7,506 (28.0)	1,456 (33.1)	609 (32.4)
Alcohol use				
Nondrinker, n (%)	8,630 (11.3)	3,175 (11.8)	715 (16.3)	305 (16.3)
Past drinker, n (%)	1,437 (1.9)	582 (2.2)	130 (3.0)	58 (3.1)
Current drinker, n (%)	57,092 (74.7)	20,145 (75.0)	3,098 (70.4)	1,327 (71.0)
Missing data, n (%)	9,281 (12.1)	2,956 (11.0)	455 (10.4)	181 (9.7)
Body mass index, mean (SD)	28.9 (5.1)	31.0 (7.7)	28.3 (5.0)	28.2 (4.9)
Missing data, n (%)	10,027 (13.1)	3,181 (11.8)	506 (11.5)	199 (10.6)
Smoking habit				
Nonsmoker, n (%)	37,834 (49.5)	12,990 (48.4)	1,985 (45.1)	847 (45.3)
Past smoker, n (%)	24,789 (32.4)	9,274 (34.5)	1,772 (40.3)	760 (40.6)
Current smoker, n (%)	10,102 (13.2)	3,478 (13.0)	477 (10.9)	199 (10.6)
Missing data, n (%)	3,715 (4.9)	1,116 (4.1)	164 (3.7)	65 (3.5)
2019 English Index of Multiple Deprivation, mean (SD)	3.0 (2.0–4.0)	3.0 (2.0–4.0)	3.0 (2.0–4.0)	3.0 (2.0–4.0)
Missing data, n (%)	8,188 (10.7)	2,455 (9.1)	104 (3.4)	42 (2.2)
Subcutaneous tophi, n (%)	361 (0.5)	12 (0.1)	37 (0.8)	5 (0.3)
Recent prior prescription for NSAIDs^e^, n (%)	5,507 (7.2)	711 (2.6)	286 (6.5)	56 (3.0)
Recent prior prescription for glucocorticoids^e^, n (%)	2,106 (2.8)	752 (2.8)	208 (4.7)	78 (4.2)
Recent prior prescription for colchicine^e^, n (%)	1,451 (1.9)	190 (0.7)	106 (2.4)	33 (0.7)
Prescription for NSAIDs on the date of the first gout consultation, n (%)	16,018 (21.0)	14,404 (39.5)	804 (18.3)	734 (39.3)
Prescription for colchicine on the date of the first gout consultation, n (%)	11,815 (15.5)	10,616 (53.6)	915 (20.8)	816 (43.6)
Hospitalization for gout on the date of the first gout consultation, n (%)	612 (0.8)	612 (2.3)	146 (3.3)	146 (7.8)
Prescription for glucocorticoids on the date of the first gout consultation, n (%)	1,313 (1.7)	1,196 (4.5)	136 (3.1)	126 (6.7)
Charlson Comorbidity Index, median (IQR)	1.0 (0.0–2.0)	1.0 (0.0–2.0)	2.0 (1.0–4.0)	3.0 (1.0–4.0)
Number of hospitalizations for any cause in the previous year, median (IQR, maximum)	0.0 (0.0–0.0, 3.0)	0.0 (0.0–0.0, 3.0)	0.0 (0.0–1.0, 3.0)	0.0 (0.0–2.0, 3.0)
Number of primary care consultations for any cause in the previous year, median (IQR)	80 (5.0–15.0)	9.0 (6.0–17.0)	15.0 (10.0–23.0)	17.0 (11.0–25.0)
ESC high/very high cardiovascular risk category,^46^ n (%)	29,754 (38.9)	11,522 (42.9)	3,707 (84.3)	1,609 (86.0)
History of acute coronary syndrome or stroke before the study start, n (%)	10,193 (13.3)	4,023 (15.0)	1,480 (33.7)	627 (33.5)
Peripheral artery disease, n (%)	3,934 (5.1)	1,593 (5.9)	643 (14.6)	283 (15.1)
Chronic kidney disease (stage III–V), n (%)	10,823 (14.2)	4,498 (16.7)	1,230 (28.0)	562 (30.1)
Chronic kidney disease (stage IV–V), n (%)	1,650 (2.2)	660 (2.5)	224 (5.1)	96 (5.1)
Diabetes mellitus, n (%)	8,490 (11.1)	3,396 (12.6)	314 (7.1)	391 (20.9)
Diabetes mellitus with target organ damage^f^, n (%)	2,619 (3.4)	1,075 (4.0)	866 (19.7)	152 (8.1)
Atrial fibrillation, n (%)	7,159 (9.4)	3,011 (11.2)	944 (21.5)	424 (22.7)
Hypercholesterolemia, n (%)	12,346 (16.2)	4,615 (17.2)	1,069 (24.3)	481 (25.7)
Arterial hypertension, n (%)	36,459 (47.7)	13,505 (50.3)	2,912 (66.2)	1,240 (66.3)
Recent prior prescription for statins^e^, n (%)	19,444 (25.4)	7,701 (28.7)	2,186 (49.7)	984 (52.6)
Recent prior prescription for antihypertensive drugs^e^, n (%)	34,003 (44.5)	12,950 (48.2)	3,111 (70.7)	1,374 (73.5)
Recent prior prescription for diuretics^e^, n (%)	25,779 (33.7)	9,599 (35.7)	2,492 (56.7)	1,060 (56.7)
Recent prior prescription for antiplatelet agents^e^, n (%)	14,307 (18.7)	5,655 (21.1)	2,268 (51.6)	1,001 (53.5)

* ESC, European Society of Cardiology; IQR, interquartile range; NSAID, nonsteroidal anti‐inflammatory drug; SCCS: self‐controlled case series.

^a^ Cohort 1 included people who met the inclusion criteria (ie, age ≥18 years old, availability of research‐quality data, two years of gout‐free registration at the current general practice, no previous hospitalizations for gout, prescriptions of urate‐lowering therapy, or mention of codes indicating prevalent gout [ie, history of gout] before or on gout diagnosis date).

^b^ Cohort 2 included people who met the inclusion criteria and were diagnosed with gout using the strict exposure definition.

^c^ SCCS 1 included people from cohort 1 who experienced at least one cardiovascular event at least two years from gout diagnosis.

^d^ SCCS 2 included people from cohort 2 who experienced at least one cardiovascular event at least two years from gout diagnosis.

^e^ A recent prior prescription was defined as a prescription of any length issued in the 60 days before the first gout diagnosis.

^f^ Target organ damage was defined according to ESC guidelines.^46^

**Table 2:** Incidence rate of cardiovascular events on days 1 to 30, 31 to 120, and 121 to 730 after first gout diagnosis*

Days after the first gout diagnosis	Number of cardiovascular events	Person‐time at risk (y)	Incidence rate per 1,000 patient‐years at risk (95% CI)	Rate difference per 1,000 patient‐years at risk (95% CI)
Patients who met the inclusion criteria were diagnosed with gout using the broad exposure definition (definition 1) (n = 76,440)				
1–30	195	6,252.2	31.2 (27.1–35.9)	Reference
31–120	426	18,367.8	23.2 (21.1–25.5)	−8.0 (−12.8 to −3.2)
121–730	2,335	109,399.6	21.3 (20.5–22.2)	−9.9 (−14.2 to −5.5)
31–730	2,761	127,767.4	21.6 (20.8–22.4)	−9.6 (−14.0 to −5.1)
Patients who met the inclusion criteria were diagnosed using the strict exposure definition (definition 2) (n = 26,858)				
1–30	66	2,195.8	30.1 (23.6–38.3)	Reference
31–120	176	6,439.4	27.3 (23.6–31.7)	−2.7 (−10.9 to 5.4)
121–730	881	38,029.0	23.2 (21.7–24.7)	−6.9 (−14.3 to 0.5)
31–730	1,057	44,468.4	23.8 (22.4–25.2)	−6.3 (−13.3 to 1.1)

* CI, confidence interval.

The incidence of cardiovascular events was significantly higher in the first 30 days after the first gout diagnosis compared with their incidence in days 121 to 730 in cohort 1 (31.2 [95% CI 27.1–35.9] vs 21.3 in [95% CI 20.5–22.2] events/1,000 person‐years) with a rate difference of −9.9 (95% CI −14.2 to −5.5) events per 1,000 person‐years. The difference was only numerically higher in cohort 2 (30.1 [95% CI 23.6–38.3] vs 23.2 [95% CI 21.7–24.7] events per 1,000 person‐years, respectively) with a rate difference of −6.9 (95% CI −14.5 to 0.5) events per 1,000 person‐years (Table 2).

### Temporal association between first recorded gout diagnosis and cardiovascular events using the broad definition (definition 1)

The 4,398 patients with a cardiovascular event within 730 days before or after gout diagnosis were included (Table 1). There were 477 and 3,921 cardiovascular events in exposed and baseline periods, respectively (aIRR 1.20, 95% CI 1.12–1.36).

**Figure 3:**
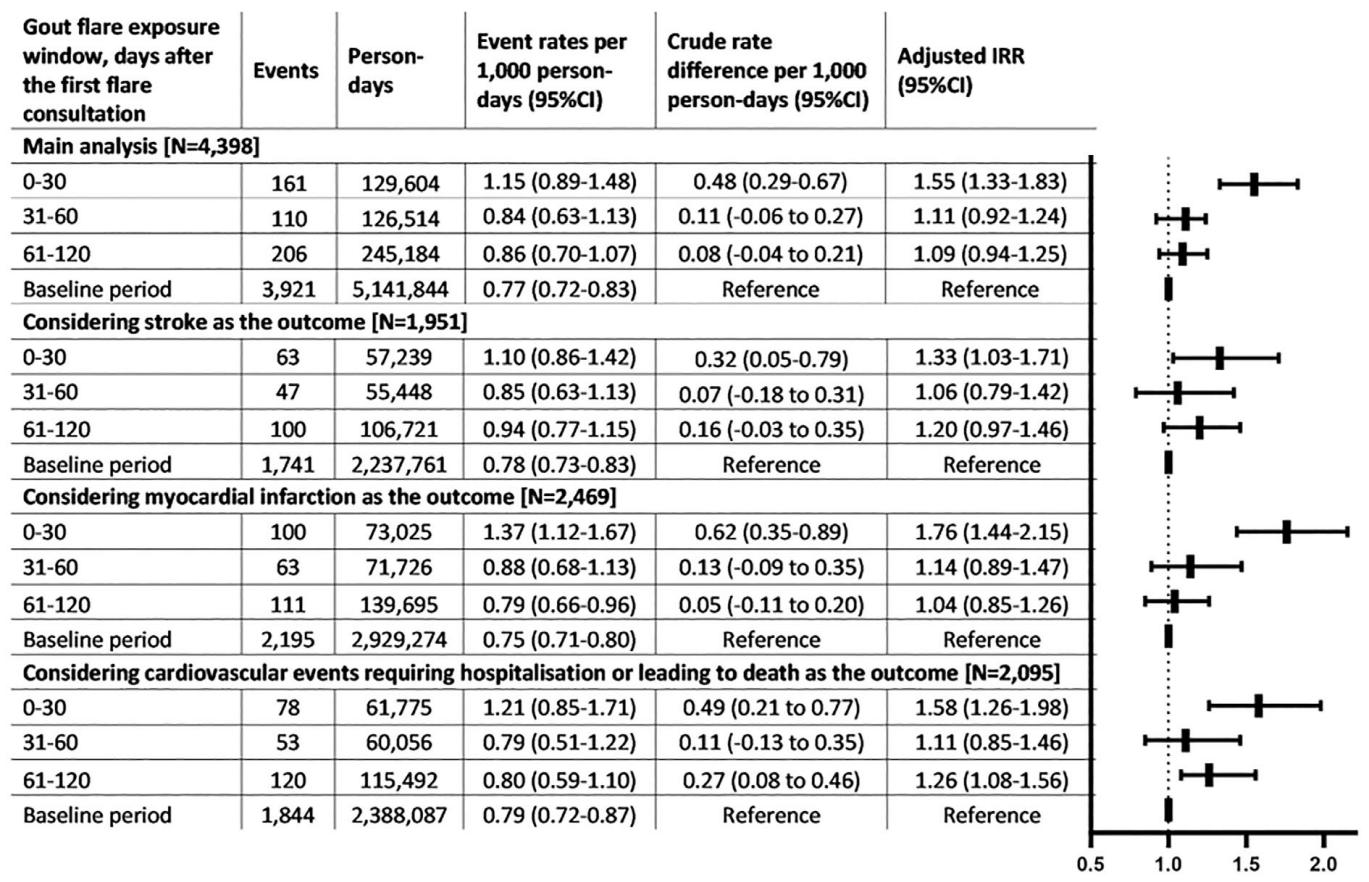
Cardiovascular events associated with first recorded diagnosis of gout in either the primary or hospitalized because of gout. The number of patients included in each analysis is reported in square brackets. The analyses were adjusted for age and calendar season. The baseline interval consisted of a pre‐exposure period of up to 730 days before the first consultation for gout and a postexposure period of up to 610 days after the exposed period at most. IRR, incidence rate ratio; CI, confidence interval.

The incidence of cardiovascular events was significantly higher in the 30 days following the first gout diagnosis. Compared with the baseline period, the aIRRs for days 0 to 30, 31 to 60, and 61 to 120 of the exposed period were 1.55 (95% CI 1.33–1.83), 1.11 (95% CI 0.92–1.34), and 1.09 (95% CI 0.94–1.25), respectively (Figure 3).

### Temporal association between the first gout diagnosis and cardiovascular events using the strict definition (definition 2)

The 1,870 patients with a cardiovascular event within 730 days before or after gout diagnosis were included in this analysis (Table 1). There were 197 and 1,673 cardiovascular events in exposed and baseline periods, respectively (aIRR 1.17, 95% CI 1.01–1.36).

**Figure 4:**
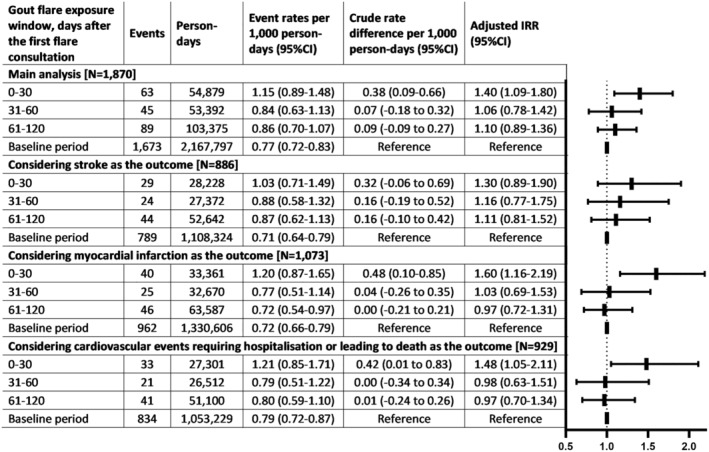
Cardiovascular events associated with the first recorded diagnosis of gout and either prescribed an anti‐inflammatory drug on the same date or hospitalized because of gout. The number of patients included in each analysis is reported in square brackets. The analyses were adjusted for age and calendar season. The baseline interval consisted of a pre‐exposure period of up to 730 days before the first consultation for gout and a postexposure period of up to 610 days after the exposed period at most. IRR, incidence rate ratio; CI, confidence interval.

The incidence of cardiovascular events was significantly higher in the 30 days following the first gout diagnosis compared with the baseline period. Compared with the baseline period, the aIRRs for days 0 to 30, 31 to 60, and 61 to 120 of the exposed period were 1.40 (95% CI 1.09–1.80), 1.06 (95% CI 0.78–1.42), and 1.10 (95% CI 0.89–1.36), respectively (Figure 4).

### Sensitivity analyses

The results of the sensitivity analyses were consistent with the main analysis (Figures 2 and 3; Supplementary Materials S4, S5, S6, and S7). The greatest relative increase was observed in the first 15 days after the first gout diagnosis (definition 1 aIRR 1.70, 95% CI 1.38–2.10; definition 2 aIRR 1.52, 95% CI 1.10–2.11). Furthermore, the temporal association was still present when we considered cardiovascular events requiring hospitalization or leading to death as the outcome (definition 1 aIRR 1.58, 95% CI 1.26–1.98; definition 2 aIRR 1.48, 95% CI 1.05–2.11) and when we excluded people with previous cardiovascular events (definition 1 aIRR 1.53, 95% CI 1.26–1.87; definition 2 aIRR 1.39, 95% CI 1.01–1.90).

There was no evidence for a statistically significant interaction between the age group and the first gout diagnosis on the occurrence of cardiovascular events (p_interaction_ = 0.14 and 0.27 for definitions 1 and 2, respectively). Women had a significantly greater increase in the risk of cardiovascular events after a new gout diagnosis than men when using a broad definition (p_interaction_ = 0.03 for definition 1) but not when using a strict definition (p_interaction_ = 0.12 for definition 2) (Supplementary Materials S5 and S7).

Results were also consistent in the stratified analyses according to comorbidities (ie, chronic kidney disease, heart failure, diabetes mellitus, and hypertension) and diuretic prescription. Although not statistically significant, the association between the first gout diagnosis and cardiovascular events was weaker in people prescribed colchicine (prescribed on the same date as the first gout diagnosis: aIRR 0.84, 95% CI 0.53–1.33; and prescribed within one week after the first gout diagnosis: aIRR 1.17, 95% CI 0.80–1.71) compared to people prescribed NSAIDs (prescribed on the same date as the first gout diagnosis: aIRR 1.39, 95% CI 0.93–2.06; prescribed within one week after the first gout diagnosis: aIRR 1.48, 95% CI 1.02–2.16) (Supplementary Material S7).

### Negative control outcome

In both SCCS analyses, the first consultation at which gout was diagnosed was not temporally associated with the first consultation for cataract (Supplementary Materials S8 and S9). Similarly, the incidence rates of the first‐ever consultation for cataract did not significantly vary between the first 30 days after the first consultation at which gout was diagnosed and days 31 to 730 in the cohort studies (Supplementary Material S10).

## DISCUSSION

We evaluated the temporal association between the first consultation at which gout was diagnosed and cardiovascular events. Using a large nationwide dataset incorporating primary care, hospitalization, and mortality records, we observed the first consultation at which gout was diagnosed to be temporally associated with a transient increase in cardiovascular events in the subsequent 30 days regardless of the definition used. Confidence in our findings was increased by the fact that the greatest relative increase was observed in the first 15 days after the first diagnosis and that the temporal association was still present when we considered hard outcomes such as cardiovascular events requiring hospitalization or leading to death. The cohort studies, including all eligible patients and showing significantly higher incidence rates of cardiovascular events in the first 30 days after the first consultation at which gout was diagnosed, were consistent with the SCCS analyses.

Previous cohort studies have shown increased cardiovascular morbidity after gout diagnosis but have not focused on early gout nor the temporal association between first gout diagnosis, which could be first gout flare consultation, and cardiovascular events.^32^, ^33^ Among people newly diagnosed with gout, the incidence of cardiovascular events within the subsequent 30 days was significantly higher than within the next two years in the present study. It was also higher than previous studies in which a longer follow‐up period after gout diagnosis was considered.^32^, ^33^

The incidence of cardiovascular events in the 30 days after the first gout diagnosis in our study was comparable to those reported in studies examining the short‐term risk of cardiovascular events following respiratory infections.^34^ Like respiratory infections, the potential mechanism underpinning the higher incidence of cardiovascular events immediately after the first gout diagnosis may be related to atherothrombosis, platelet activation, endothelial dysfunction, and biomechanical stress triggered by systemic and vascular inflammation during gout flares.^35^ Several mediators such as interleukin‐1beta, interleukin‐6, and tumor necrosis factor superfamily 14 are elevated during gout flares and have been shown to promote both cardiovascular events^36^, ^39^, ^40^ and local and systemic inflammation.^41^ Further proof of their role comes from the observation that the inhibition of interleukin‐1beta by canakinumab and inhibition of inflammasome assembly by colchicine prevents cardiovascular events in populations without gout.^36^, ^37^, ^38^

In a previous study that included people with long‐standing gout, we reported that gout flares were associated with a transient increase in cardiovascular events.^5^ Lopez et al observed that the first‐ever hospital admission for gout flare was associated with an increased risk of major acute cardiovascular events (ie, acute coronary syndrome, stroke, heart failure, or cardiovascular death) in the 30 days following the hospital discharge in an Australian population of 941 patients with gout.^6^ However, the generalizability of the latter study was limited by hospital‐based recruitment. Those who may seek hospital admission for gout flare may either have severe and/or refractory gout flare and comorbidities that make it difficult for them to be managed in primary care. Moreover, neither of these two studies assessed risk following the first gout diagnosis. In the present study, we also used two definitions to ascertain the first gout diagnosis. The broad definition allowed us to capture all the consultations at which gout was diagnosed in primary care and secondary care. Because flares are by far the most common presentation of gout,^1^ we speculated that any first record of gout can be reasonably considered as a proxy for the first gout flare requiring contact with the health care system. The strict definition enabled us to establish the internal validity of the association because it captured all the consultations at which the first gout flare was recorded in primary care and secondary care.

Although not statistically significant, women had a higher incidence rate of cardiovascular events after the diagnosis of gout compared to men. This finding is in line with a recently published paper^2^ in which the excess risk of a broad range of cardiovascular diseases in gout was greater in women than men. This could be related to the older age and the higher burden of cardiovascular comorbidities, such as obesity, dyslipidemia, chronic kidney disease, diabetes mellitus, and heart failure.^42^

The main strength of this study is the use of SCCS design that allows us to examine the association between a transient exposure and an outcome of interest automatically accounting for fixed covariates. As recommended, we checked the validity of the assumptions. We extracted data from a high‐quality, nationwide, general population–based electronic health record database containing both primary care, secondary care, and mortality data, allowing us to capture all outcomes. We also estimate the absolute incidence of cardiovascular events in people with a new diagnosis of gout, showing a similar temporal trend. Thus, our results are generalizable. Also, the use of routinely collected data allowed us to minimize the risk of recall bias. Selection bias was minimized by universal access to health care for all UK residents free at the point of use. The lack of a temporal association with a negative control outcome supports a low risk of unmeasured and unmeasurable bias, such as the health care‐seeking behavior bias.

We must acknowledge some limitations. The first is the uncertainty regarding the onset of gout symptoms. We used the date of the contact with the health care system on which a gout diagnosis was made as the exposure date. We believe that it is a reasonable proxy, especially in the context of gout flares in which symptoms usually peak within 12 to 24 hours of onset. We could not be sure about the fact that the first gout diagnosis is the consultation for the first‐ever gout flare experienced by the patient. However, gout flare has typical characteristics, and it is likely that most patients in this study were consulting for gout flare for the first time.^43^

Although we performed sensitivity, stratified, and negative control analyses, another limitation is that the risk of residual unmeasured time‐varying confounding could not be completely ruled out. However, results of the analyses stratified on the major cardiovascular risk factors for gout flares (ie, chronic kidney disease, diabetes mellitus, arterial hypertension, heart failure, and prescription of diuretics) were consistent with the main analysis. Third, we did not conduct an adjudication of cardiovascular outcomes. However, these outcomes have been widely adopted in previous research.^23^, ^24^ Fourth, these results might apply only to gout flares that are of sufficient severity to require contact with the health care system. Fifth, the available sample size limited our ability to draw any definite conclusion on the comparative impact of different anti‐inflammatory medications prescribed to treat gout flares. Nevertheless, patients who received a prescription for colchicine had a nonsignificant decrease in the incidence of cardiovascular events in the first 30 days compared to those prescribed with NSAIDs or glucocorticoids. Sixth, we cannot exclude that a proportion of patients with gout can be misdiagnosed with other crystal arthropathies, osteoarthritis, or other chronic inflammatory arthritis. However, previous validation studies have shown that a primary care diagnosis of gout has a positive predictive value of 90%.^44^, ^45^ Furthermore, the association was still significant when we considered those with serum urate levels ≥480 μmol/L. Seventh, the prevalence of tophaceous gout was relatively low. This may be related to the fact that the study population consisted of people with early gout. However, it is possible that the low prevalence was because of a poor recording of subcutaneous tophi in primary care. Finally, our dataset did not include data about consultations for gout in accident and emergency departments that did not require overnight admission to the hospital. Although such patients may have more severe gout flares than those who only consulted their general practitioner, they would have a less severe gout flare and fewer comorbidities than those requiring overnight hospital admission; we do not believe that their exclusion biases the observed association.

The first gout diagnosis was associated with an increased risk of cardiovascular events in the short term. Inflammation during the first gout flare is the most plausible explanation for this association. These findings support the need for cardiovascular risk management from the time of first gout consultation.

## AUTHOR CONTRIBUTIONS

All authors contributed to at least one of the following manuscript preparation roles: conceptualization AND/OR methodology, software, investigation, formal analysis, data curation, visualization, and validation AND drafting or reviewing/editing the final draft. As corresponding author, Dr Cipolletta confirms that all authors have provided the final approval of the version to be published, and takes responsibility for the affirmations regarding article submission (eg, not under consideration by another journal), the integrity of the data presented, and the statements regarding compliance with institutional review board/Helsinki Declaration requirements.

## Supporting information


Disclosure form

**Appendix S1:** Supplementary Materials
