# Supplementary material for: Short‐Term Risk of Cardiovascular Events in People Newly Diagnosed With Gout
Source: Arthritis Rheumatol. 2024 Oct 17;77(2):202–11. doi: 10.1002/art.42986 (PMC11782110; doi:10.1002/art.42986)
Supplement: Supplementary file 2 — Appendix S1: Supplementary Materials [file ART-77-202-s002.docx]

**Supplementary Materials**

Supplementary Material S1 page 2

Supplementary Material S2 page 3

Supplementary Material S3 page 6

Supplementary Material S4 page 28

Supplementary Material S5 page 29

Supplementary Material S6 page 31

Supplementary Material S7 page 32

Supplementary Material S8 page 34

Supplementary Material S9 page 35

Supplementary Material S10 page 36

References page 37

**Supplementary Material S1**

A total of 96,153 incident gout patients were ascertained in the CPRD between 1997 and 2020 in an earlier study [1]. Based on previous data, we assumed an annual incidence rate of cardiovascular events ranging between 19 and 43 per 1000 person-years in gout [2,3] and half of patients meeting our strict definition for gout flare (definition 2, i.e., first gout consultation with treatment prescribed on the same date in primary-care, first gout consultation with specific codes indicating acute arthritis or hospitalization with gout as the first diagnosis recorded on discharge summary on the date of the first gout consultation).

Considering an exposed period of 120 days, a baseline period of 1340 days, and 100% of cases exposed during the SCCS study period, the SCCS analysis had 90% power to detect an incidence rate ratio associated with the exposure of 1.4 with a type I error of 2.5% provided there were at least 1265 cardiovascular events in the SCCS study period.

**Supplementary Material S2**

The SCCS assumptions could be violated by outcomes which increase mortality in the short term or when the outcome of interest influences the likelihood of exposure [4,5].

To evaluate if event-dependent exposure could be an issue for the SCCS analyses, we plotted the number of outcomes by time before or after gout diagnosis using both definitions for gout flare (Supplementary Figure S1 and Supplementary Figure S2).

**Supplementary Figure S1**. Number of cardiovascular events over the study period in the SCCS analysis which included people with newly diagnosed gout consulting for their first gout flare according to the broad definition (definition 1).


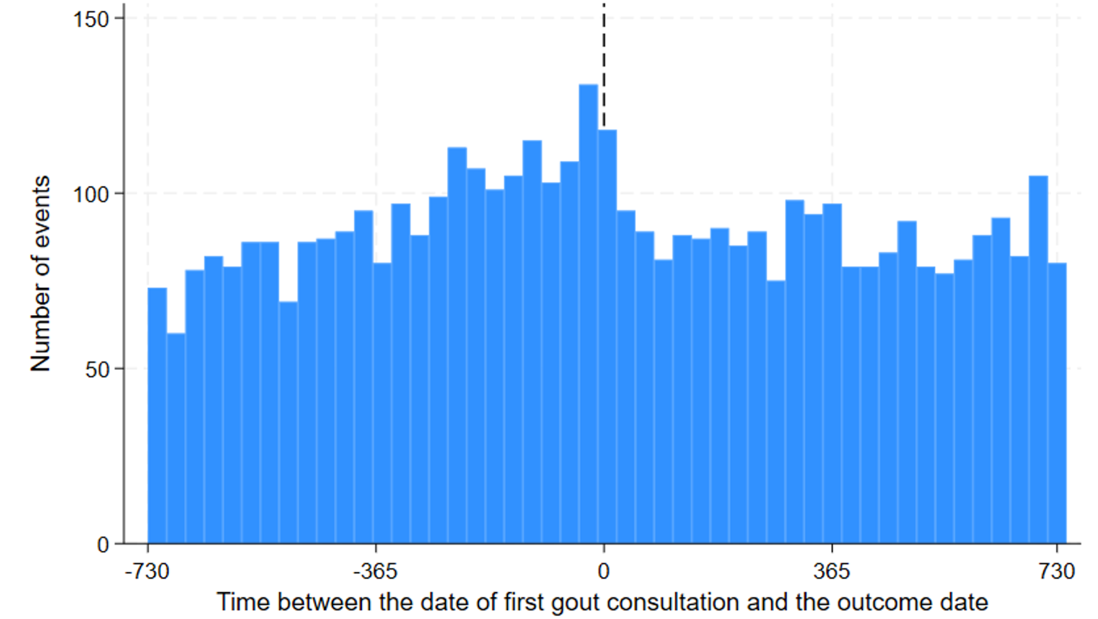


**Supplementary Figure S2**. Number of cardiovascular events over the study period in the SCCS analysis which included people with newly diagnosed gout consulting for their first gout flare according to the strict definition (definition 2).


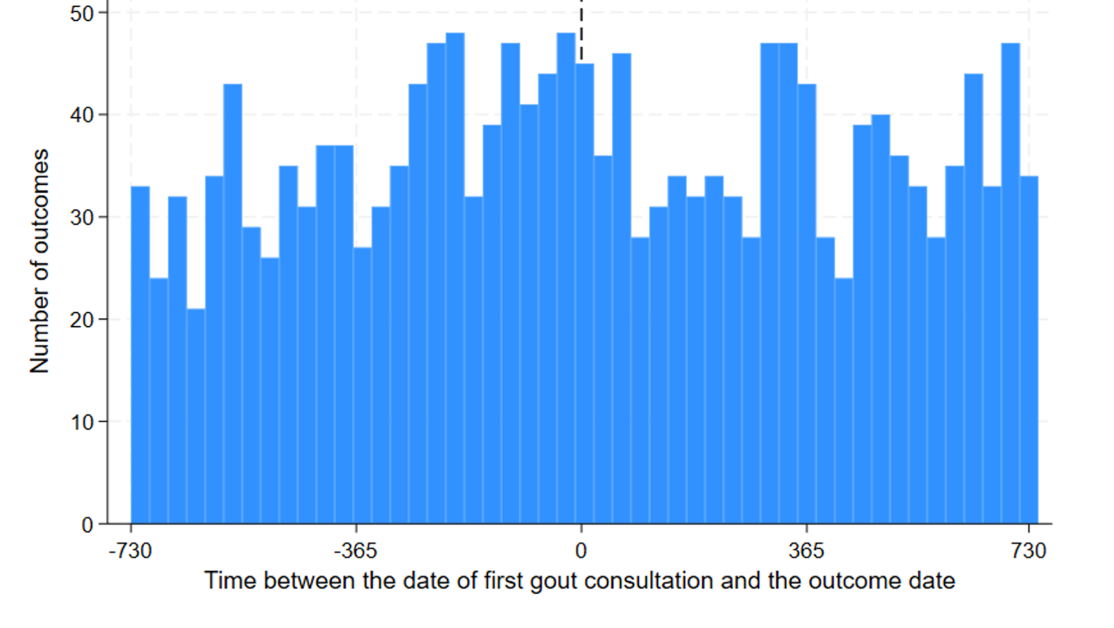


We did not observe a decrease in the occurrence of outcomes in the 30 days immediately before gout diagnosis. Therefore, an induction period was not added to the analyses.

To assess if event-dependent observation periods could be an issue for the analyses, we plotted a histogram of the time from the outcome to the actual end of observation in patients who were censored and uncensored using both definitions for gout flare (Supplementary Figure S3 and Supplementary Figure S4).

**Supplementary Figure S3**. Time between the outcome date and the end of the study or the censored date in people who died due to the cardiovascular event or were censored on the date of the next gout flare and in uncensored people in the SCCS analysis which included people with newly diagnosed gout consulting for their first gout flare according to the broad definition (definition 1).


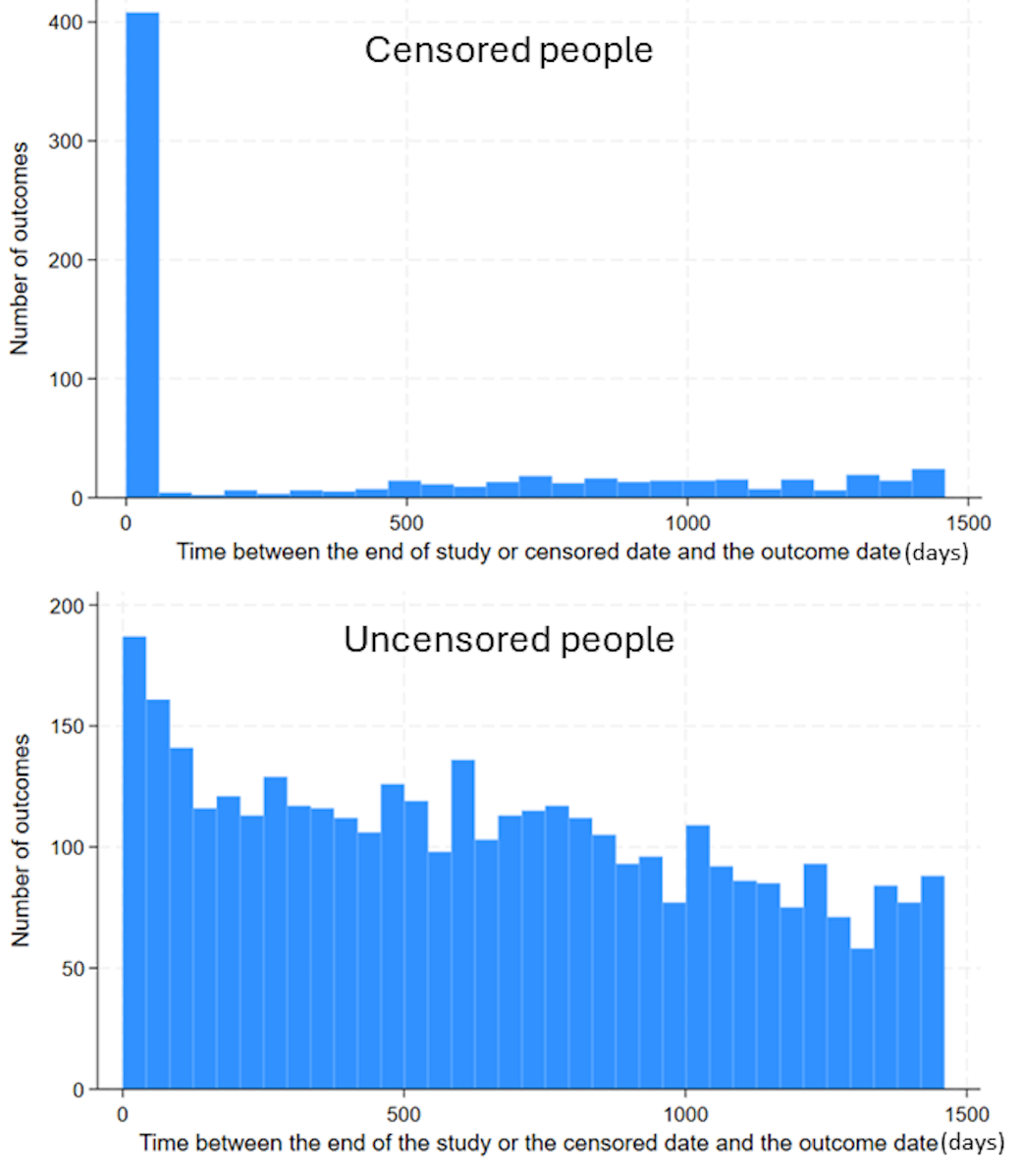


**Supplementary Figure S4**. Time between the outcome date and the end of the study or the censored date in people who died due to the cardiovascular event or were censored on the date of the next gout flare and in uncensored people in the SCCS analysis which included people with newly diagnosed gout consulting for their first gout flare according to the strict definition (definition 2).


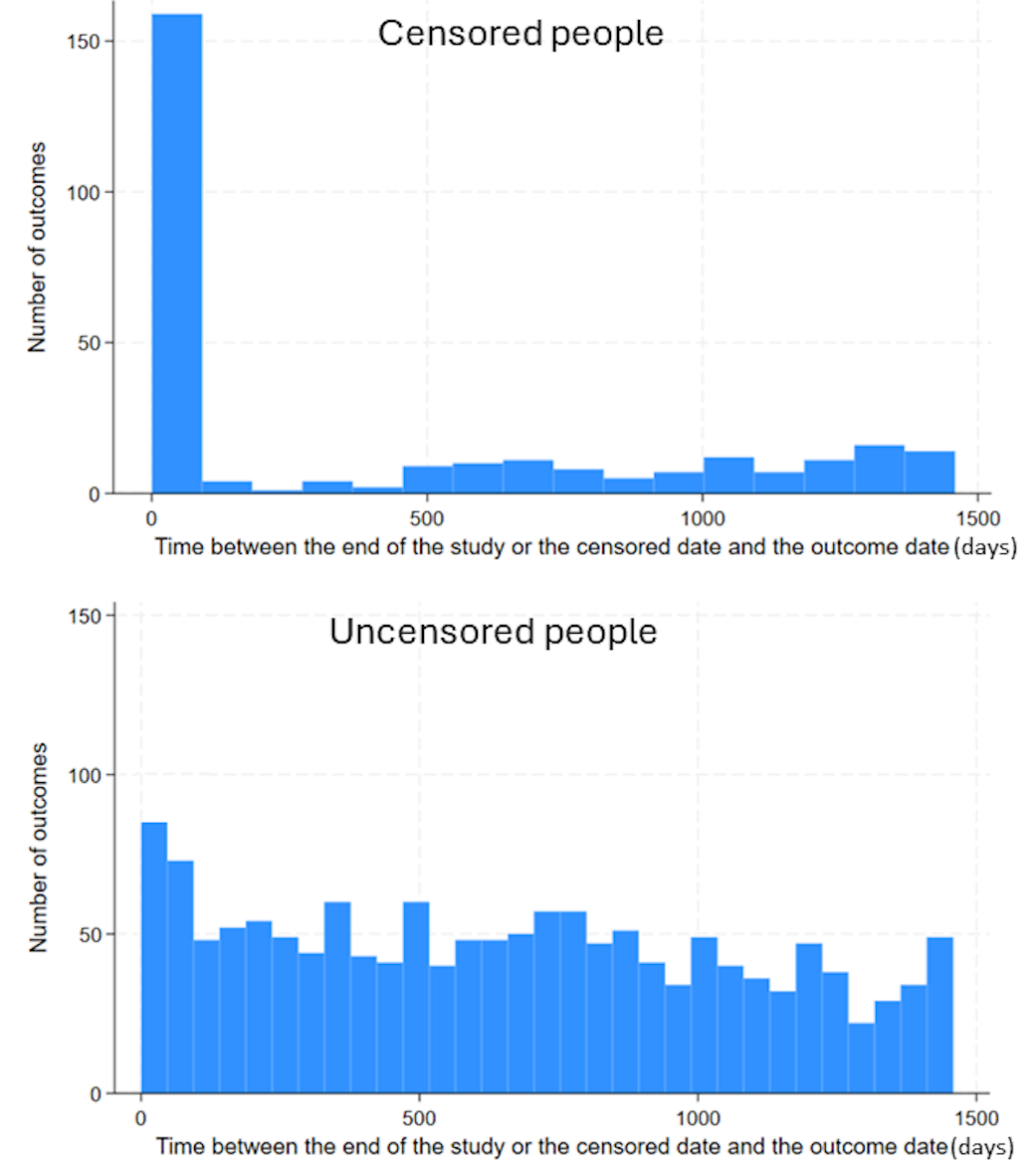


A spike close to zero is apparent in the censored data histograms. This finding indicates the presence of event-dependent observation periods (censoring on the date of death due to outcome), which we tested further with sensitivity analyses.

**Supplementary Material S3**

**Incident gout codes**

| **Medcode** | **Read term** |
| --- | --- |
| 709 | Gout |
| 2857 | Gouty arthritis |
| 4440 | Gouty tophi of other sites |
| 9874 | Gouty tophi of hand |
| 10080 | Gouty arthropathy |
| 11462 | Idiopathic gout |
| 12594 | Gouty arthritis NOS |
| 14996 | Initial gout assessment |
| 17284 | O/E - auricle of ear - tophi |
| 21687 | Gout due to impairment of renal function |
| 24153 | Gout NOS |
| 27521 | Other specified gouty manifestation NOS |
| 28999 | Other specified gouty manifestation |
| 29658 | Joints gout affected |
| 35664 | Gouty arthritis of the ankle and foot |
| 36481 | Gouty tophi of ear |
| 45465 | Gouty arthritis of the forearm |
| 49775 | Gouty arthritis of the lower leg |
| 52101 | Gouty arthritis of the hand |
| 58064 | Gouty arthritis of multiple sites |
| 58746 | Gout associated problems |
| 60541 | Gouty arthritis of other specified site |
| 72471 | Gouty arthritis of the shoulder region |
| 93677 | Gouty arthritis of toe |
| 94539 | [X]Other secondary gout |
| 97539 | Gouty arthritis of the upper arm |
| 108901 | Acute exacerbation of gout |
| 113314 | Gouty arthritis of unspecified site |
| 59344 | Gouty neuritis |
| **Gout flare codes** | |
| **Medcode** | **Read term** |
| 45465 | Gouty arthritis of the forearm |
| 12594 | Gouty arthritis NOS |
| 97539 | Gouty arthritis of the upper arm |
| 2857 | Gouty arthritis |
| 93677 | Gouty arthritis of toe |
| 60541 | Gouty arthritis of other specified site |
| 52101 | Gouty arthritis of the hand |
| 58064 | Gouty arthritis of multiple sites |
| 108901 | Acute exacerbation of gout |
| 35664 | Gouty arthritis of the ankle and foot |
| 49775 | Gouty arthritis of the lower leg |
| 72471 | Gouty arthritis of the shoulder region |
| 113314 | Gouty arthritis of unspecified site |
| **ICD-10** | **Term** |
| M10 | Gout |

**Prevalent gout codes**

| **Medcode** | **Read term** |
| --- | --- |
| 3759 | H/O: gout |
| 16475 | Gout monitoring |
| 34006 | Date gout treatment started |
| 34105 | Gout treatment changed |
| 35660 | Follow-up gout assessment |
| 43646 | Date gout treatment stopped |
| 52117 | Gout monitoring NOS |
| 68209 | Date of last gout attack |
| 52103 | Gout drug side effects |

**Urate-lowering therapies codes**

| **Prodcode** | **Substance name** | **BNF code** |
| --- | --- | --- |
| 11975 | Allopurinol | 10010451/10010452 |
| 34005 | Allopurinol | 10010451 |
| 41612 | Allopurinol | 10010451 |
| 23368 | Allopurinol | 10010451/10010452 |
| 46941 | Allopurinol | 10010451/10010452 |
| 44239 | Allopurinol | 10010451/10010452 |
| 368 | Allopurinol | 10010451 |
| 19037 | Allopurinol | 10010451 |
| 78470 | Allopurinol | 10010451 |
| 64906 | Allopurinol | 10010451 |
| 41520 | Allopurinol | 10010451 |
| 34566 | Allopurinol | 10010451 |
| 67256 | Allopurinol | 10010451 |
| 72223 | Allopurinol | 10010451 |
| 34711 | Allopurinol | 10010451 |
| 44240 | Allopurinol | 10010451/10010452 |
| 24215 | Allopurinol | 10010451 |
| 67748 | Allopurinol | 10010451 |
| 34930 | Allopurinol | 10010451 |
| 30768 | Allopurinol | 10010451 |
| 19201 | Allopurinol | 10010451 |
| 33484 | Allopurinol | 10010451 |
| 34947 | Allopurinol | 10010451/10010452 |
| 71008 | Allopurinol | 10010451 |
| 74645 | Allopurinol | 10010451/10010452 |
| 41541 | Allopurinol | 10010451 |
| 76 | Allopurinol | 10010451 |
| 34278 | Allopurinol | 10010451 |
| 77467 | Allopurinol | 10010451 |
| 34573 | Allopurinol | 10010451 |
| 68025 | Allopurinol | 10010451 |
| 13467 | Allopurinol | 10010451 |
| 76324 | Allopurinol | 10010451 |
| 7805 | Allopurinol | 10010451 |
| 413 | Allopurinol | 10010451 |
| 41664 | Allopurinol | 10010451/10010452 |
| 71717 | Allopurinol | 10010451 |
| 78521 | Allopurinol | 10010451 |
| 5182 | Allopurinol | 10010451 |
| 17255 | Allopurinol | 10010451/10010452 |
| 76616 | Allopurinol | 10010451/10010452 |
| 72153 | Allopurinol | 10010451 |
| 45352 | Allopurinol | 10010451 |
| 83504 | Allopurinol | 10010451 |
| 42859 | Febuxostat | 10010451/10010452 |
| 43336 | Febuxostat | 10010451/10010452 |
| 42536 | Febuxostat | 10010451/10010452 |
| 43161 | Febuxostat | 10010451/10010452 |
| 77342 | Benzbromarone | 10010000 |
| 47263 | Benzbromarone | 10010000 |
| 31662 | Probenecid | 10010451 |
| 68886 | Probenecid | 10010451 |
| 8944 | Probenecid | 10010451/10010452 |
| 1302 | Probenecid | 10010451 |
| 5204 | Sulfinpyrazone | 10010451 |
| 20133 | Sulfinpyrazone | 10010451 |
| 18519 | Sulfinpyrazone | 10010451 |
| 9951 | Sulfinpyrazone | 10010451 |
| 13419 | Sulfinpyrazone | 10010451 |
| 10554 | Sulfinpyrazone | 10010451 |

**Colchicine codes**

| **Prodcode** | **Substance name** | **BNF code** |
| --- | --- | --- |
| 77032 | Colchicine | 10010450 |
| 76421 | Colchicine | 10010450 |
| 34469 | Colchicine | 10010450 |
| 34329 | Colchicine | 10010450 |
| 71853 | Colchicine | 10010450 |
| 83462 | Colchicine | 10010450 |
| 762 | Colchicine | 10010450 |
| 46962 | Colchicine | 10010450 |
| 66469 | Colchicine | 10010450 |
| 71052 | Colchicine | 10010450 |
| 8643 | Colchicine | 0 |
| 12086 | Colchicine | 0 |
| 80866 | Colchicine | 0 |
| 357 | Colchicine | 0 |
| 21063 | Colchicine | 0 |

**Non-steroidal anti-inflammatory codes**

| **Prodcode** | **Substance name** | **BNF code** |
| --- | --- | --- |
| 526 | Aceclofenac | 10010100 |
| 9474 | Aceclofenac | 10010100 |
| 72925 | Celecoxib | 10010100 |
| 5254 | Celecoxib | 10010100 |
| 55582 | Celecoxib | 10010100 |
| 474 | Celecoxib | 10010100 |
| 66571 | Celecoxib | 10010100 |
| 50059 | Celecoxib | 10010100 |
| 72293 | Celecoxib | 10010100 |
| 65016 | Celecoxib | 10010100 |
| 64245 | Celecoxib | 10010100 |
| 66757 | Celecoxib | 10010100 |
| 64935 | Celecoxib | 10010100 |
| 43616 | Celecoxib | 10010100 |
| 62840 | Celecoxib | 10010100 |
| 52420 | Celecoxib | 10010100 |
| 77553 | Celecoxib | 10010100 |
| 80017 | Celecoxib | 10010100 |
| 81403 | Celecoxib | 10010100 |
| 80671 | Celecoxib | 10010100 |
| 5080 | Celecoxib | 10010100 |
| 11907 | Dexibuprofen | 10010100 |
| 21421 | Dexibuprofen | 10010100 |
| 10325 | Dexibuprofen | 10010100 |
| 21419 | Dexibuprofen | 10010100 |
| 9637 | Dexketoprofen trometamol | 10010100 |
| 5173 | Dexketoprofen trometamol | 10010100 |
| 50602 | Diclofenac potassium | 10010100 |
| 51293 | Diclofenac potassium | 10010100 |
| 48059 | Diclofenac potassium | 10010100 |
| 5085 | Diclofenac potassium | 10010100 |
| 52338 | Diclofenac potassium | 10010100 |
| 69477 | Diclofenac potassium | 10010100 |
| 597 | Diclofenac potassium | 10010100 |
| 53345 | Diclofenac potassium | 10010100 |
| 65783 | Diclofenac potassium | 10010100 |
| 58071 | Diclofenac potassium | 10010100 |
| 51099 | Diclofenac potassium | 10010100 |
| 70468 | Diclofenac potassium | 10010100 |
| 82690 | Diclofenac potassium | 10010100 |
| 43045 | Diclofenac potassium | 10010100 |
| 70145 | Diclofenac potassium | 10010100 |
| 33669 | Diclofenac sodium | 10010100/10010450 |
| 29037 | Diclofenac sodium | 10010100 |
| 82517 | Diclofenac sodium | 10010100 |
| 17532 | Diclofenac sodium | 10010100 |
| 2386 | Diclofenac sodium | 10010100 |
| 50058 | Diclofenac sodium | 10010100 |
| 57162 | Diclofenac sodium | 10010100 |
| 27055 | Diclofenac sodium | 10010100 |
| 73109 | Diclofenac sodium | 10010100 |
| 67220 | Diclofenac sodium | 10010100 |
| 42793 | Diclofenac sodium | 10010100/10010450 |
| 16286 | Diclofenac sodium | 10010100 |
| 14084 | Diclofenac sodium | 10010100 |
| 59880 | Diclofenac sodium | 10010100 |
| 54660 | Diclofenac Sodium | 10010100/10010450 |
| 18798 | Diclofenac sodium | 10010100 |
| 9886 | Diclofenac sodium | 10010100/10010450 |
| 17491 | Diclofenac sodium | 10010100/10010450 |
| 25283 | Diclofenac sodium | 10010100/10010450 |
| 14672 | Diclofenac sodium | 10010100 |
| 71064 | Diclofenac sodium | 10010100 |
| 19382 | Diclofenac sodium | 10010100 |
| 21610 | Diclofenac sodium | 10010100 |
| 75442 | Diclofenac sodium | 10010100 |
| 4506 | Diclofenac sodium | 10010100 |
| 34744 | Diclofenac sodium | 10010100/10010450 |
| 56898 | Diclofenac sodium | 10010100 |
| 60443 | Diclofenac sodium | 10010100 |
| 47501 | Diclofenac sodium | 10010100 |
| 26165 | Diclofenac sodium | 10010100 |
| 71362 | Diclofenac sodium | 10010100 |
| 48218 | Diclofenac sodium | 10010100/10010450 |
| 33645 | Diclofenac sodium | 10010100/10010450 |
| 1984 | Diclofenac Sodium | 10010100/10010450 |
| 31383 | Diclofenac sodium | 10010100 |
| 3421 | Diclofenac sodium | 10010100/10010450 |
| 39823 | Diclofenac sodium | 10010100 |
| 33559 | Diclofenac sodium | 10010100/10010450 |
| 74028 | Diclofenac sodium | 10010100 |
| 20384 | Diclofenac sodium | 10010100 |
| 31787 | Diclofenac sodium | 10010100 |
| 1446 | Diclofenac sodium | 10010100/10010450 |
| 14707 | Diclofenac sodium | 10010100 |
| 34271 | Diclofenac sodium | 10010100 |
| 33457 | Diclofenac sodium | 10010100/10010450 |
| 29330 | Diclofenac sodium | 10010100 |
| 34212 | Diclofenac sodium | 10010100/10010450 |
| 50317 | Diclofenac sodium | 10010100 |
| 57045 | Diclofenac sodium | 10010100 |
| 33994 | Diclofenac sodium | 10010100 |
| 17030 | Diclofenac sodium | 10010100 |
| 26351 | Diclofenac sodium | 10010100 |
| 8062 | Diclofenac sodium | 10010100 |
| 32854 | Diclofenac sodium | 10010100 |
| 447 | Diclofenac sodium | 10010100 |
| 79845 | Diclofenac sodium | 10010100 |
| 30942 | Diclofenac sodium | 10010100/10010450 |
| 1115 | Diclofenac sodium | 10010100 |
| 61596 | Diclofenac sodium | 10010100 |
| 74048 | Diclofenac sodium | 10010100 |
| 78675 | Diclofenac sodium | 10010100 |
| 66577 | Diclofenac sodium | 10010100 |
| 30297 | Diclofenac sodium | 10010100/10010450 |
| 34487 | Diclofenac sodium | 10010100 |
| 56078 | Diclofenac sodium | 10010100 |
| 21824 | Diclofenac sodium | 10010100 |
| 28553 | Diclofenac sodium | 10010100 |
| 42455 | Diclofenac sodium | 10010100 |
| 72396 | Diclofenac sodium | 10010100 |
| 17128 | Diclofenac sodium | 10010100 |
| 580 | Diclofenac sodium | 10010100 |
| 74835 | Diclofenac sodium | 10010100 |
| 18371 | Diclofenac sodium | 10010100/10010450 |
| 49059 | Diclofenac sodium | 10010100 |
| 30282 | Diclofenac sodium | 10010100/10010450 |
| 27200 | Diclofenac sodium | 10010100 |
| 1075 | Diclofenac Sodium | 10010100/10010450 |
| 16272 | Diclofenac sodium | 10010100 |
| 31950 | Diclofenac sodium | 10010100 |
| 83653 | Diclofenac sodium | 10010100 |
| 42406 | Diclofenac sodium | 10010100/10010450 |
| 32916 | Diclofenac sodium | 10010100/10010450 |
| 1233 | Diclofenac Sodium | 10010100/10010450 |
| 4692 | Diclofenac sodium | 10010100 |
| 68354 | Diclofenac sodium | 10010100 |
| 8789 | Diclofenac sodium | 10010100/10010450 |
| 77405 | Diclofenac sodium | 10010100 |
| 71117 | Diclofenac sodium | 10010100 |
| 72546 | Diclofenac sodium | 10010100 |
| 81751 | Diclofenac sodium | 10010100 |
| 24236 | Diclofenac sodium | 10010100/10010450 |
| 71100 | Diclofenac sodium | 10010100 |
| 9500 | Diclofenac sodium | 10010100 |
| 26888 | Diclofenac sodium | 10010100/10010450 |
| 53384 | Diclofenac sodium | 10010100 |
| 25358 | Diclofenac sodium | 10010100 |
| 31589 | Diclofenac sodium | 10010100 |
| 38948 | Diclofenac sodium | 10010100 |
| 35893 | Diclofenac sodium | 10010100 |
| 11322 | Diclofenac sodium | 10010100/10010450 |
| 54463 | Diclofenac sodium | 10010100/10010450 |
| 77392 | Diclofenac sodium | 10010100 |
| 30849 | Diclofenac sodium | 10010100 |
| 42905 | Diclofenac sodium | 10010100/10010450 |
| 15732 | Diclofenac sodium | 10010100 |
| 24122 | Diclofenac sodium | 10010100 |
| 46844 | Diclofenac sodium | 10010100 |
| 69584 | Diclofenac sodium | 10010100 |
| 10917 | Diclofenac sodium | 10010100 |
| 9222 | Diclofenac sodium | 10010100 |
| 50785 | Diclofenac sodium | 10010100 |
| 28764 | Diclofenac sodium | 10010100/10010450 |
| 29181 | Diclofenac sodium | 10010100 |
| 66123 | Diclofenac sodium | 10010100 |
| 54021 | Diclofenac sodium | 10010100 |
| 74211 | Diclofenac sodium | 10010100 |
| 27362 | Diclofenac sodium | 10010100/10010450 |
| 16225 | Diclofenac sodium | 10010100/10010450 |
| 81801 | Diclofenac sodium | 10010100 |
| 417 | Diclofenac sodium | 10010100 |
| 589 | Diclofenac sodium | 10010100 |
| 4631 | Diclofenac sodium | 10010100 |
| 59289 | Diclofenac sodium | 10010100 |
| 4625 | Diclofenac sodium | 10010100 |
| 30790 | Diclofenac sodium | 10010100/10010450 |
| 59595 | Diclofenac sodium | 10010100 |
| 3852 | Diclofenac sodium | 10010100/10010450 |
| 21387 | Diclofenac sodium | 10010100 |
| 30806 | Diclofenac sodium | 10010100/10010450 |
| 14085 | Diclofenac sodium | 10010100 |
| 17525 | Diclofenac sodium | 10010100 |
| 38881 | Diclofenac sodium | 10010100 |
| 55099 | Diclofenac sodium | 10010100 |
| 20621 | Diclofenac sodium | 10010100 |
| 17126 | Diclofenac sodium | 10010100 |
| 917 | Diclofenac Sodium | 10010100/10010450 |
| 28256 | Diclofenac sodium | 10010100/10010450 |
| 58048 | Diclofenac sodium | 10010100 |
| 3416 | Diclofenac sodium | 10010100 |
| 40086 | Diclofenac sodium | 10010100 |
| 17124 | Diclofenac sodium | 10010100/10010450 |
| 20395 | Diclofenac sodium | 10010100 |
| 39264 | Diclofenac sodium | 10010100 |
| 26631 | Diclofenac sodium | 10010100 |
| 17029 | Diclofenac sodium | 10010100 |
| 54518 | Diclofenac sodium | 10010100 |
| 1766 | Diclofenac sodium | 10010100/10010450 |
| 2904 | Diclofenac sodium | 10010100 |
| 36486 | Diclofenac sodium | 10010100 |
| 60666 | Diclofenac sodium | 10010100 |
| 40 | Diclofenac sodium | 10010100 |
| 65877 | Diclofenac sodium | 10010100 |
| 15201 | Diclofenac sodium | 10010100 |
| 9465 | Diclofenac sodium | 10010100/10010450 |
| 38992 | Diclofenac sodium | 10010100 |
| 29455 | Diclofenac sodium | 10010100 |
| 60786 | Diclofenac sodium | 10010100 |
| 71307 | Diclofenac sodium | 10010100 |
| 11168 | Diclofenac sodium | 10010100 |
| 16222 | Diclofenac sodium | 10010100 |
| 20805 | Diclofenac sodium | 10010100 |
| 9688 | Diclofenac sodium | 10010100 |
| 54075 | Diclofenac sodium | 10010100 |
| 2387 | Diclofenac sodium/Misoprostol | 10010100 |
| 81644 | Diclofenac sodium/Misoprostol | 10010100 |
| 64595 | Diclofenac sodium/Misoprostol | 10010100 |
| 162 | Diclofenac sodium/Misoprostol | 10010100 |
| 82550 | Diclofenac sodium/Misoprostol | 10010100 |
| 50269 | Diclofenac sodium/Misoprostol | 10010100 |
| 71088 | Diclofenac sodium/Misoprostol | 10010100 |
| 4880 | Diclofenac sodium/Misoprostol | 10010100 |
| 1692 | Diclofenac sodium/Misoprostol | 10010100 |
| 58842 | Diclofenac sodium/Misoprostol | 10010100 |
| 58415 | Diclofenac sodium/Misoprostol | 10010100 |
| 65528 | Diclofenac sodium/Misoprostol | 10010100 |
| 70438 | Diclofenac sodium/Misoprostol | 10010100 |
| 78096 | Etoricoxib | 10010100 |
| 53576 | Etoricoxib | 10010100 |
| 9822 | Etoricoxib | 10010100 |
| 81837 | Etoricoxib | 10010100 |
| 78072 | Etoricoxib | 10010100 |
| 74952 | Etoricoxib | 10010100 |
| 82301 | Etoricoxib | 10010100 |
| 66486 | Etoricoxib | 10010100 |
| 82428 | Etoricoxib | 10010100 |
| 5812 | Etoricoxib | 10010100 |
| 5938 | Etoricoxib | 10010100 |
| 650 | Etoricoxib | 10010100 |
| 62843 | Etoricoxib | 10010100 |
| 62658 | Etoricoxib | 10010100 |
| 64521 | Etoricoxib | 10010100 |
| 62251 | Etoricoxib | 10010100 |
| 82181 | Etoricoxib | 10010100 |
| 80442 | Etoricoxib | 10010100 |
| 6464 | Etoricoxib | 10010100 |
| 81720 | Etoricoxib | 10010100 |
| 56584 | Etoricoxib | 10010100 |
| 83341 | Etoricoxib | 10010100 |
| 51284 | Etoricoxib | 10010100 |
| 83636 | Etoricoxib | 10010100 |
| 6498 | Etoricoxib | 10010100 |
| 10589 | Fenoprofen calcium | 0 |
| 4469 | Fenoprofen calcium | 10010100 |
| 4564 | Fenoprofen Calcium | 10010100 |
| 17754 | Fenoprofen Calcium | 10010100 |
| 4565 | Fenoprofen calcium | 0 |
| 10678 | Fenoprofen calcium | 10010100 |
| 18527 | Ibuprofen | 10010100 |
| 66544 | Ibuprofen | 10010100 |
| 83943 | Ibuprofen | 10010100 |
| 21815 | Ibuprofen | 10010100 |
| 55009 | Ibuprofen | 10010100 |
| 31469 | Ibuprofen | 10010100 |
| 34536 | Ibuprofen | 10010100 |
| 21821 | Ibuprofen | 10010100 |
| 54137 | Ibuprofen | 10010100 |
| 14333 | Ibuprofen | 10010100 |
| 21045 | Ibuprofen | 10010100 |
| 34961 | Ibuprofen | 10010100 |
| 78149 | Ibuprofen | 10010100 |
| 1086 | Ibuprofen | 10010100 |
| 37648 | Ibuprofen | 10010100 |
| 65025 | Ibuprofen | 10010100 |
| 76041 | Ibuprofen | 10010100 |
| 59562 | Ibuprofen | 10010100 |
| 4911 | Ibuprofen | 10010100 |
| 849 | Ibuprofen | 10010100 |
| 4216 | Ibuprofen | 10010100 |
| 29068 | Ibuprofen | 10010100 |
| 48546 | Ibuprofen | 10010100 |
| 66648 | Ibuprofen | 10010100 |
| 27782 | Ibuprofen | 10010100 |
| 1739 | Ibuprofen | 10010100 |
| 39019 | Ibuprofen | 10010100 |
| 77335 | Ibuprofen | 10010100 |
| 57112 | Ibuprofen | 10010100 |
| 55233 | Ibuprofen | 10010100 |
| 32875 | Ibuprofen | 10010100 |
| 56213 | Ibuprofen | 10010100 |
| 48644 | Ibuprofen | 10010100 |
| 8401 | Ibuprofen | 10010100 |
| 17201 | Ibuprofen | 10010100 |
| 3599 | Ibuprofen | 10010100 |
| 58652 | Ibuprofen | 10010100 |
| 25619 | Ibuprofen | 10010100 |
| 21813 | Ibuprofen | 10010100 |
| 71041 | Ibuprofen | 10010100 |
| 11980 | Ibuprofen | 10010100 |
| 55434 | Ibuprofen | 10010100 |
| 56039 | Ibuprofen | 10010100 |
| 36606 | Ibuprofen | 10010100 |
| 33589 | Ibuprofen | 10010100 |
| 79167 | Ibuprofen | 10010100 |
| 82137 | Ibuprofen | 10010100 |
| 37253 | Ibuprofen | 10010100 |
| 29587 | Ibuprofen | 10010100 |
| 19046 | Ibuprofen | 10010100 |
| 15068 | Ibuprofen | 10010100 |
| 34729 | Ibuprofen | 10010100 |
| 43911 | Ibuprofen | 10010100 |
| 1392 | Ibuprofen | 10010100 |
| 15 | Ibuprofen | 10010100 |
| 25794 | Ibuprofen | 10010100 |
| 34550 | Ibuprofen | 10010100 |
| 63079 | Ibuprofen | 10010100 |
| 73743 | Ibuprofen | 10010100 |
| 71968 | Ibuprofen | 10010100 |
| 45216 | Ibuprofen | 10010100 |
| 74313 | Ibuprofen | 10010100 |
| 63036 | Ibuprofen | 10010100 |
| 27783 | Ibuprofen | 10010100 |
| 32100 | Ibuprofen | 10010100 |
| 50628 | Ibuprofen | 10010100 |
| 34889 | Ibuprofen | 10010100 |
| 40394 | Ibuprofen | 10010100 |
| 41701 | Ibuprofen | 10010100 |
| 67740 | Ibuprofen | 10010100 |
| 34425 | Ibuprofen | 10010100 |
| 32242 | Ibuprofen | 10010100 |
| 67594 | Ibuprofen | 10010100 |
| 75677 | Ibuprofen | 10010100 |
| 32365 | Ibuprofen | 10010100 |
| 43456 | Ibuprofen | 10010100 |
| 46921 | Ibuprofen | 10010100 |
| 74806 | Ibuprofen | 10010100 |
| 50314 | Ibuprofen | 10010100 |
| 29316 | Ibuprofen | 10010100 |
| 24305 | Ibuprofen | 10010100 |
| 45842 | Ibuprofen | 10010100 |
| 34757 | Ibuprofen | 10010100 |
| 27968 | Ibuprofen | 10010100 |
| 37553 | Ibuprofen | 10010100 |
| 46942 | Ibuprofen | 10010100 |
| 2129 | Ibuprofen | 10010100 |
| 2622 | Ibuprofen | 0 |
| 16193 | Ibuprofen | 0 |
| 407 | Ibuprofen | 10010100 |
| 18364 | Ibuprofen | 10010100 |
| 29524 | Ibuprofen | 10010100 |
| 82503 | Ibuprofen | 10010100 |
| 34359 | Ibuprofen | 10010100 |
| 39873 | Ibuprofen | 10010100 |
| 55313 | Ibuprofen | 10010100 |
| 73433 | Ibuprofen | 10010100 |
| 40253 | Ibuprofen | 10010100 |
| 36787 | Ibuprofen lysine | 04070100/10010100 |
| 55153 | Ibuprofen lysine | 10010100 |
| 54514 | Ibuprofen lysine | 10010100 |
| 33935 | Ibuprofen lysine | 04070100/10010100 |
| 26095 | Ibuprofen lysine | 10010100 |
| 44483 | Ibuprofen sodium dihydrate | 10010100 |
| 66567 | Ibuprofen sodium dihydrate | 10010100 |
| 78779 | Ketoprofen | 10010100/10010450 |
| 3326 | Ketoprofen | 10010100/10010450 |
| 77293 | Ketoprofen | 10010100 |
| 30327 | Ketoprofen | 10010100 |
| 33568 | Ketoprofen | 10010100/10010450 |
| 17818 | Ketoprofen | 10010100 |
| 40141 | Ketoprofen | 10010100 |
| 13347 | Ketoprofen | 10010100/10010450 |
| 3043 | Ketoprofen | 10010100 |
| 40484 | Ketoprofen | 10010100 |
| 46920 | Ketoprofen | 10010100/10010450 |
| 12122 | Ketoprofen | 10010100/10010450 |
| 7840 | Ketoprofen | 10010100/10010450 |
| 40215 | Ketoprofen | 10010100 |
| 74005 | Ketoprofen | 10010100 |
| 33180 | Ketoprofen | 10010100/10010450 |
| 71104 | Ketoprofen | 10010100 |
| 40185 | Ketoprofen | 10010100 |
| 11999 | Ketoprofen | 10010100/10010450 |
| 1231 | Ketoprofen | 10010100 |
| 389 | Ketoprofen | 10010100 |
| 31916 | Ketoprofen | 10010100 |
| 18647 | Ketoprofen | 10010100 |
| 57943 | Ketoprofen | 10010100 |
| 25701 | Ketoprofen | 10010100 |
| 21955 | Ketoprofen | 10010100 |
| 8385 | Ketoprofen | 0 |
| 21050 | Ketoprofen | 10010100/10010450 |
| 75573 | Ketoprofen | 10010100 |
| 838 | Ketoprofen | 10010100/10010450 |
| 46919 | Ketoprofen | 10010100/10010450 |
| 32227 | Ketoprofen | 10010100 |
| 42500 | Ketoprofen | 10010100/10010450 |
| 75771 | Ketoprofen | 10010100/10010450 |
| 71376 | Ketoprofen | 10010100 |
| 31962 | Ketoprofen | 10010100 |
| 10336 | Ketoprofen | 0 |
| 75581 | Ketoprofen | 10010100/10010450 |
| 7432 | Ketoprofen | 10010100 |
| 27013 | Ketoprofen | 10010100/10010450 |
| 77459 | Ketoprofen | 10010100 |
| 71127 | Ketoprofen | 10010100 |
| 40664 | Ketoprofen | 10010100 |
| 29772 | Ketoprofen | 10010100 |
| 67803 | Ketoprofen | 10010100 |
| 27082 | Ketoprofen | 10010100 |
| 1571 | Ketoprofen | 10010100 |
| 46940 | Ketoprofen | 10010100 |
| 83807 | Ketoprofen | 10010100/10010450 |
| 40336 | Ketoprofen | 10010100 |
| 15286 | Ketoprofen | 10010100 |
| 24111 | Ketorolac trometamol | 0 |
| 16637 | Ketorolac trometamol | 15010402 |
| 21949 | Ketorolac trometamol | 15010402 |
| 10939 | Ketorolac trometamol | 15010402 |
| 14251 | Ketorolac trometamol | 15010402 |
| 3336 | Ketorolac trometamol | 15010402 |
| 59139 | Ketorolac trometamol | 15010402 |
| 30122 | Lornoxicam | 10010100 |
| 29110 | Lornoxicam | 0 |
| 28383 | Lumiracoxib | 0 |
| 7118 | Lumiracoxib | 0 |
| 10212 | Lumiracoxib | 0 |
| 28171 | Lumiracoxib | 0 |
| 33801 | Mefenamic acid | 10010100 |
| 46967 | Mefenamic acid | 10010100 |
| 1073 | Mefenamic acid | 10010100 |
| 34595 | Mefenamic acid | 10010100 |
| 57007 | Mefenamic acid | 10010100 |
| 32090 | Mefenamic acid | 10010100 |
| 57297 | Mefenamic acid | 10010100 |
| 32105 | Mefenamic acid | 10010100 |
| 34898 | Mefenamic acid | 10010100 |
| 13459 | Mefenamic acid | 10010100 |
| 36260 | Mefenamic acid | 10010100 |
| 259 | Mefenamic acid | 10010100 |
| 51827 | Mefenamic acid | 10010100 |
| 26522 | Mefenamic acid | 10010100 |
| 296 | Mefenamic acid | 10010100 |
| 34910 | Mefenamic acid | 10010100 |
| 1983 | Mefenamic Acid | 10010100 |
| 1246 | Mefenamic Acid | 10010100 |
| 26247 | Mefenamic acid | 10010100 |
| 22230 | Mefenamic acid | 10010100 |
| 79173 | Mefenamic acid | 10010100 |
| 4710 | Mefenamic acid | 10010100 |
| 34924 | Mefenamic acid | 10010100 |
| 48810 | Mefenamic acid | 10010100 |
| 46968 | Mefenamic acid | 10010100 |
| 32234 | Mefenamic acid | 10010100 |
| 64103 | Mefenamic acid | 10010100 |
| 21831 | Mefenamic acid | 10010100 |
| 30389 | Mefenamic acid | 10010100 |
| 34793 | Mefenamic acid | 10010100 |
| 41524 | Mefenamic acid | 10010100 |
| 34438 | Mefenamic acid | 10010100 |
| 66452 | Mefenamic acid | 10010100 |
| 41677 | Mefenamic acid | 10010100 |
| 126 | Mefenamic acid | 10010100 |
| 30391 | Mefenamic acid | 10010100 |
| 75569 | Mefenamic acid | 10010100 |
| 61581 | Mefenamic acid | 10010100 |
| 70221 | Mefenamic acid | 10010100 |
| 35935 | Meloxicam | 10010100 |
| 56275 | Meloxicam | 10010100 |
| 71989 | Meloxicam | 10010100 |
| 77260 | Meloxicam | 10010100 |
| 1470 | Meloxicam | 10010100 |
| 57475 | Meloxicam | 10010100 |
| 850 | Meloxicam | 10010100 |
| 66364 | Meloxicam | 10010100 |
| 2243 | Meloxicam | 10010100 |
| 79307 | Meloxicam | 10010100 |
| 68932 | Meloxicam | 10010100 |
| 83175 | Meloxicam | 10010100 |
| 78629 | Meloxicam | 10010100 |
| 61235 | Meloxicam | 10010100 |
| 60705 | Meloxicam | 10010100 |
| 76191 | Meloxicam | 10010100 |
| 83496 | Meloxicam | 10010100 |
| 1469 | Meloxicam | 10010100 |
| 57370 | Meloxicam | 10010100 |
| 41365 | Omeprazole/Ketoprofen | 01030500/10010100 |
| 41364 | Omeprazole/Ketoprofen | 01030500/10010100 |
| 41367 | Omeprazole/Ketoprofen | 01030500/10010100 |
| 41366 | Omeprazole/Ketoprofen | 01030500/10010100 |
| 51306 | Parecoxib sodium | 15010402 |
| 50080 | Parecoxib sodium | 15010402 |
| 54760 | Parecoxib sodium | 15010402 |
| 570 | Parecoxib Sodium | 15010402 |
| 58644 | Parecoxib sodium | 15010402 |
| 19975 | Parecoxib Sodium | 15010402 |
| 43541 | Piroxicam | 10010100 |
| 71027 | Piroxicam | 10010100 |
| 41623 | Piroxicam | 10010100 |
| 82354 | Piroxicam | 10010100 |
| 29465 | Piroxicam | 10010100 |
| 1755 | Piroxicam | 10010100 |
| 67608 | Piroxicam | 10010100 |
| 3710 | Piroxicam | 10010100 |
| 3409 | Piroxicam | 10010100 |
| 7524 | Piroxicam | 10010100 |
| 31777 | Piroxicam | 10010100 |
| 67815 | Piroxicam | 10010100 |
| 41624 | Piroxicam | 10010100 |
| 2827 | Piroxicam | 0 |
| 2463 | Piroxicam | 0 |
| 141 | Piroxicam | 10010100 |
| 19320 | Piroxicam | 10010100 |
| 21864 | Piroxicam | 10010100 |
| 77185 | Piroxicam | 10010100 |
| 41621 | Piroxicam | 10010100 |
| 44703 | Piroxicam | 10010100 |
| 341 | Piroxicam | 10010100 |
| 3935 | Piroxicam | 10010100 |
| 74659 | Piroxicam | 10010100 |
| 39109 | Piroxicam | 10010100 |
| 26234 | Piroxicam | 10010100 |
| 37750 | Piroxicam | 10010100 |
| 21846 | Piroxicam | 10010100 |
| 73981 | Piroxicam | 10010100 |
| 77694 | Piroxicam | 10010100 |
| 27490 | Piroxicam | 0 |
| 28695 | Piroxicam | 10010100 |
| 41622 | Piroxicam | 10010100 |
| 21123 | Piroxicam | 10010100 |
| 4965 | Piroxicam | 10010100 |
| 83492 | Piroxicam | 10010100 |
| 27484 | Piroxicam | 0 |
| 10169 | Piroxicam betadex | 10010100 |
| 11495 | Piroxicam betadex | 10010100 |
| 706 | Rofecoxib | 0 |
| 666 | Rofecoxib | 0 |
| 518 | Rofecoxib | 0 |
| 67786 | Rofecoxib | 0 |
| 6460 | Rofecoxib | 0 |
| 538 | Rofecoxib | 0 |
| 5841 | Rofecoxib | 0 |
| 53145 | Rofecoxib | 0 |
| 5695 | Rofecoxib | 0 |
| 640 | Rofecoxib | 0 |
| 637 | Rofecoxib | 0 |
| 613 | Rofecoxib | 0 |
| 5739 | Rofecoxib | 0 |
| 12075 | Tenoxicam | 10010100 |
| 42604 | Tenoxicam | 10010100 |
| 31064 | Tenoxicam | 10010100 |
| 71152 | Tenoxicam | 10010100 |
| 47816 | Tenoxicam | 10010100 |
| 3974 | Tenoxicam | 10010100 |
| 24531 | Tenoxicam | 10010100 |
| 17572 | Tenoxicam | 10010100 |
| 24682 | Tenoxicam | 10010100 |
| 28332 | Tenoxicam | 10010100 |
| 387 | Tiaprofenic acid | 0 |
| 20059 | Tiaprofenic Acid | 10010100 |
| 7913 | Tiaprofenic acid | 0 |
| 14776 | Tiaprofenic acid | 10010100 |
| 2257 | Tiaprofenic acid | 0 |
| 2382 | Tiaprofenic acid | 0 |
| 1778 | Tiaprofenic acid | 10010100 |
| 2863 | Tiaprofenic acid | 10010100 |
| 25643 | Tiaprofenic Acid | 10010100 |
| 9912 | Valdecoxib | 0 |
| 9978 | Valdecoxib | 0 |
| 18066 | Valdecoxib | 0 |
| 9899 | Valdecoxib | 0 |
| 723 | Valdecoxib | 0 |
| 6663 | Valdecoxib | 0 |
| 1049 | Aspirin | 02090000/04070100 |
| 9939 | Aspirin | 02090000/04070100 |
| 11977 | Aspirin | 02090000/04070100 |
| 36521 | Aspirin | 02090000/04070100 |
| 41766 | Aspirin | 02090000/04070100 |
| 657 | Aspirin | 02090000/04070100 |
| 1902 | Aspirin | 02090000/04070100 |
| 12976 | Aspirin DL-Lysine/Metoclopramide hydroc | 4070100 |
| 5288 | Aspirin DL-Lysine/Metoclopramide hydroc | 4070100 |

**Corticosteroids codes**

| **Prodcode** | **Substance name** | **BNF code** |
| --- | --- | --- |
| 251655001000027000 | Dexamethasone | 6030200 |
| 24580511000001100 | Dexamethasone | 03020000/04065300/06030200 |
| 8792611000001100 | Dexamethasone | 03020000/04065300/06030200 |
| 30114311000001100 | Prednisolone | 01050200/03020000/05011000/05040800/06030200/08020200/10010201 |
| 512811000001108 | Prednisolone | 01050200/03020000/05011000/05040800/06030200/08020200/10010201 |
| 18307211000001100 | Prednisolone | 01050200/03020000/05011000/05040800/06030200/08020200/10010201 |
| 17916811000001100 | Prednisolone | 01050200/03020000/05011000/05040800/06030200/08020200/10010201 |
| 17916411000001100 | Prednisolone | 01050200/03020000/05011000/05040800/06030200/08020200/10010201 |
| 22520311000001100 | Prednisolone | 01050200/03020000/05011000/05040800/06030200/08020200/10010201 |
| 29785001000027100 | Prednisolone | 6030200 |
| 32584711000001100 | Prednisolone | 01050200/03020000/05011000/05040800/06030200/08020200/10010201 |
| 779211000001109 | Prednisolone | 01050200/03020000/05011000/05040800/06030200/08020200/10010201 |
| 459611000001100 | Prednisolone | 01050200/03020000/05011000/05040800/06030200/08020200/10010201 |
| 325443009 | Prednisolone | 01050200/03020000/05011000/05040800/06030200/08020200/10010201 |
| 33425811000001100 | Prednisolone | 01050200/03020000/05011000/05040800/06030200/08020200/10010201 |
| 19743211000001100 | Prednisolone | 01050200/03020000/05011000/05040800/06030200/08020200/10010201 |
| 662011000001105 | Prednisolone | 01050200/03020000/05011000/05040800/06030200/08020200/10010201 |
| 19743011000001100 | Prednisolone | 01050200/03020000/05011000/05040800/06030200/08020200/10010201 |
| 22453011000001100 | Prednisolone | 01050200/03020000/05011000/05040800/06030200/08020200/10010201 |
| 33577911000001100 | Prednisolone | 01050200/03020000/05011000/05040800/06030200/08020200/10010201 |
| 858811000001104 | Prednisolone | 01050200/03020000/05011000/05040800/06030200/08020200/10010201 |
| 17999611000001100 | Prednisolone | 01050200/03020000/05011000/05040800/06030200/08020200/10010201 |
| 32652511000001100 | Prednisolone | 01050200/03020000/05011000/05040800/06030200/08020200/10010201 |
| 17999311000001100 | Prednisolone | 01050200/03020000/05011000/05040800/06030200/08020200/10010201 |
| 331111000001100 | Prednisolone | 01050200/03020000/05011000/05040800/06030200/08020200/10010201 |
| 255211000001101 | Prednisolone | 01050200/03020000/05011000/05040800/06030200/08020200/10010201 |
| 22519711000001100 | Prednisolone | 01050200/03020000/05011000/05040800/06030200/08020200/10010201 |
| 37130211000001100 | Prednisolone | 01050200/03020000/05011000/05040800/06030200/08020200/10010201 |
| 380011000001100 | Prednisolone | 01050200/03020000/05011000/05040800/06030200/08020200/10010201 |
| 325442004 | Prednisolone | 01050200/03020000/05011000/05040800/06030200/08020200/10010201 |
| 32611811000001100 | Prednisolone | 01050200/03020000/05011000/05040800/06030200/08020200/10010201 |
| 29795001000027100 | Prednisolone | 6030200 |
| 22452711000001100 | Prednisolone | 01050200/03020000/05011000/05040800/06030200/08020200/10010201 |
| 32652311000001100 | Prednisolone | 01050200/03020000/05011000/05040800/06030200/08020200/10010201 |
| 33428411000001100 | Prednisolone | 01050200/03020000/05011000/05040800/06030200/08020200/10010201 |
| 175775001000027000 | Dexamethasone Sodium Phosphate | 06030200/10010202 |
| 78075001000027100 | Dexamethasone Sodium Phosphate | 06030200/10010202 |
| 104285001000027000 | Dexamethasone Sodium Phosphate | 06030200/10010202 |
| 65615001000027100 | Methylprednisolone Acetate | 06030200/10010202 |
| 125255001000027000 | Methylprednisolone Acetate | 06030200/10010202 |
| 77745001000027100 | Prednisolone Sodium Phosphate | 06030200/10010202 |
| 65635001000027100 | Lidocaine Hydrochloride/Methylprednisolone Acetate | 06030200/10010202 |
| 78065001000027100 | Dexamethasone Sodium Phosphate | 06030200/10010202 |
| 150805001000027000 | Lidocaine Hydrochloride/Methylprednisolone Acetate | 06030200/10010202 |
| 100755001000027000 | Dexamethasone Sodium Phosphate | 06030200/10010202 |
| 109225001000027000 | Dexamethasone Sodium Phosphate | 06030200/10010202 |
| 67855001000027100 | Dexamethasone Sodium Phosphate | 06030200/10010202 |
| 109245001000027000 | Dexamethasone Sodium Phosphate | 06030200/10010202 |
| 109235001000027000 | Dexamethasone Sodium Phosphate | 06030200/10010202 |
| 17873911000001100 | Prednisone | 6030200 |
| 17872911000001100 | Prednisone | 6030200 |
| 17873811000001100 | Prednisone | 6030200 |
| 17872811000001100 | Prednisone | 6030200 |
| 17873311000001100 | Prednisone | 6030200 |
| 17873211000001100 | Prednisone | 6030200 |
| 19792911000001100 | Dexamethasone sodium phosphate | 0 |
| 429995001 | Prednisolone | 01050200/03020000/05011000/05040800/06030200/08020200/10010201/10020100 |
| 30129111000001100 | Prednisolone | 01050200/03020000/05011000/05040800/06030200/08020200/10010201/10020100 |
| 8452411000001100 | Dexamethasone | 03020000/04065300/06030200 |
| 19792911000001100 | Dexamethasone sodium phosphate | 03020000/04065300/06030200 |
| 196715001000027000 | Dexamethasone | 6030200 |
| 11715411000001100 | Dexamethasone | 03020000/04065300/06030200 |
| 13079111000001100 | Prednisolone | 01050200/03020000/05011000/05040800/06030200/08020200/10010201/10020100 |
| 29361211000001100 | Prednisolone | 03020000/06030200/10010201 |
| 23590911000001100 | Dexamethasone sodium phosphate | 03020000/04065300/06030200 |
| 31386211000001100 | Prednisolone | 03020000/06030200/10010201 |
| 13079511000001100 | Prednisolone | 01050200/03020000/05011000/05040800/06030200/08020200/10010201/10020100 |
| 13079311000001100 | Prednisolone | 01050200/03020000/05011000/05040800/06030200/08020200/10010201/10020100 |
| 29424511000001100 | Prednisolone | 03020000/06030200/10010201 |
| 11612111000001100 | Dexamethasone | 03020000/04065300/06030200 |
| 11716011000001100 | Dexamethasone | 03020000/04065300/06030200 |
| 13078911000001100 | Prednisolone | 01050200/03020000/05011000/05040800/06030200/08020200/10010201/10020100 |
| 28995411000001100 | Prednisolone | 03020000/06030200/10010201 |
| 28876811000001100 | Dexamethasone sodium phosphate | 03020000/04065300/06030200 |
| 10528411000001100 | Dexamethasone sodium phosphate | 03020000/04065300/06030200 |
| 61165001000027100 | Dexamethasone | 6030200 |
| 24350911000001100 | Dexamethasone sodium phosphate | 03020000/04065300/06030200 |
| 8452111000001100 | Dexamethasone | 03020000/04065300/06030200 |
| 325365005 | Dexamethasone | 03020000/04065300/06030200 |
| 29559211000001100 | Prednisolone | 03020000/06030200/10010201 |
| 11716411000001100 | Dexamethasone | 03020000/04065300/06030200 |
| 3851611000001100 | Dexamethasone sodium phosphate | 03020000/04065300/06030200 |
| 28881311000001100 | Dexamethasone sodium phosphate | 03020000/04065300/06030200 |
| 13078311000001100 | Prednisolone | 01050200/03020000/05011000/05040800/06030200/08020200/10010201/10020100 |
| 13133111000001100 | Prednisolone | 03020000/06030200/10010201 |
| 21926211000001100 | Dexamethasone sodium phosphate | 03020000/04065300/06030200 |
| 11716211000001100 | Dexamethasone | 03020000/04065300/06030200 |
| 37084211000001100 | Dexamethasone sodium phosphate | 03020000/04065300/06030200 |
| 32688311000001100 | Prednisolone | 03020000/06030200/10010201 |
| 416533002 | Prednisolone | 01050200/03020000/05011000/05040800/06030200/08020200/10010201/10020100 |
| 11714511000001100 | Dexamethasone | 03020000/04065300/06030200 |
| 29904211000001100 | Prednisolone | 01050200/03020000/05011000/05040800/06030200/08020200/10010201/10020100 |
| 8429111000001100 | Dexamethasone | 03020000/04065300/06030200 |
| 432224007 | Prednisolone | 01050200/03020000/05011000/05040800/06030200/08020200/10010201/10020100 |
| 11715711000001100 | Dexamethasone | 03020000/04065300/06030200 |
| 13079611000001100 | Prednisolone | 01050200/03020000/05011000/05040800/06030200/08020200/10010201/10020100 |
| 11714911000001100 | Dexamethasone | 03020000/04065300/06030200 |
| 8429011000001100 | Dexamethasone | 03020000/04065300/06030200 |
| 8452511000001100 | Dexamethasone | 03020000/04065300/06030200 |
| 11716511000001100 | Dexamethasone | 03020000/04065300/06030200 |
| 13005411000001100 | Methylprednisolone | 6030200 |
| 8452211000001100 | Dexamethasone | 03020000/04065300/06030200 |
| 13079211000001100 | Prednisolone | 01050200/03020000/05011000/05040800/06030200/08020200/10010201/10020100 |
| 13078411000001100 | Prednisolone | 01050200/03020000/05011000/05040800/06030200/08020200/10010201/10020100 |
| 13079411000001100 | Prednisolone | 01050200/03020000/05011000/05040800/06030200/08020200/10010201/10020100 |
| 13078611000001100 | Prednisolone | 01050200/03020000/05011000/05040800/06030200/08020200/10010201/10020100 |
| 11714611000001100 | Dexamethasone | 03020000/04065300/06030200 |
| 11716111000001100 | Dexamethasone | 03020000/04065300/06030200 |
| 8452311000001100 | Dexamethasone | 03020000/04065300/06030200 |
| 33037911000001100 | Deflazacort | 6030200 |
| 11714811000001100 | Dexamethasone | 03020000/04065300/06030200 |
| 11716311000001100 | Dexamethasone | 03020000/04065300/06030200 |
| 4392411000001100 | Methylprednisolone sodium succinate | 06030200/10010202 |
| 4396711000001100 | Methylprednisolone sodium succinate | 06030200/10010202 |
| 4396911000001100 | Methylprednisolone sodium succinate | 06030200/10010202 |
| 4393011000001100 | Methylprednisolone sodium succinate | 06030200/10010202 |
| 34743111000001100 | Dexamethasone sodium phosphate | 06030200/64090000 |
| 34035411000001100 | Prednisolone sodium phosphate | 01050200/03020000/05011000/05040800/06030200/08020200/10010201 |
| 3694811000001100 | Betamethasone sodium phosphate | 6030200 |
| 21229111000001100 | Betamethasone sodium phosphate | 6030200 |
| 34743411000001100 | Dexamethasone sodium phosphate | 06030200/64090000 |
| 157711000001100 | Prednisolone sodium phosphate | 01050200/03020000/05011000/05040800/06030200/08020200/10010201 |
| 19280911000001100 | Betamethasone sodium phosphate | 6030200 |
| 32400911000001100 | Dexamethasone sodium phosphate | 06030200/64090000 |
| 21142211000001100 | Betamethasone sodium phosphate | 6030200 |
| 32401311000001100 | Dexamethasone sodium phosphate | 06030200/64090000 |
| 245011000001108 | Prednisolone sodium phosphate | 01050200/03020000/05011000/05040800/06030200/08020200/10010201 |
| 299285001000027000 | Prednisolone sodium phosphate | 6030200 |
| 21232511000001100 | Betamethasone sodium phosphate | 6030200 |
| 392511000001108 | Prednisolone sodium phosphate | 01050200/03020000/05011000/05040800/06030200/08020200/10010201 |
| 28937511000001100 | Prednisolone sodium phosphate | 01050200/03020000/05011000/05040800/06030200/08020200/10010201 |
| 32072011000001100 | Dexamethasone sodium phosphate | 06030200/64090000 |
| 39722711000001100 | Prednisolone sodium phosphate | 01050200/03020000/05011000/05040800/06030200/08020200/10010201 |
| 25675001000027100 | Prednisolone sodium phosphate | 6030200 |
| 39697311000001100 | Betamethasone sodium phosphate | 6030200 |
| 30114611000001100 | Prednisolone sodium phosphate | 01050200/03020000/05011000/05040800/06030200/08020200/10010201 |
| 32071911000001100 | Dexamethasone sodium phosphate | 06030200/64090000 |
| 32071811000001100 | Dexamethasone sodium phosphate | 06030200/64090000 |
| 34172711000001100 | Prednisolone sodium phosphate | 01050200/03020000/05011000/05040800/06030200/08020200/10010201 |
| 4052611000001100 | Dexamethasone sodium phosphate | 06030200/10010202 |
| 28040011000001100 | Dexamethasone sodium phosphate | 06030200/10010202 |
| 4052511000001100 | Dexamethasone sodium phosphate | 06030200/10010202 |
| 27957911000001100 | Dexamethasone sodium phosphate | 06030200/10010202 |
| 3932011000001100 | Betamethasone sodium phosphate | 06030200/10010202 |
| 4052811000001100 | Dexamethasone sodium phosphate | 6030200 |
| 4052711000001100 | Dexamethasone sodium phosphate | 06030200/10010202 |
| 4045311000001100 | Dexamethasone sodium phosphate | 06030200/10010202 |
| 4043411000001100 | Dexamethasone sodium phosphate | 6030200 |
| 4044311000001100 | Dexamethasone sodium phosphate | 06030200/10010202 |
| 35910611000001100 | Betamethasone sodium phosphate | 06030200/10010202 |
| 22153811000001100 | Dexamethasone sodium phosphate | 06030200/10010202 |
| 19532111000001100 | Dexamethasone sodium phosphate | 06030200/10010202 |
| 9154711000001100 | Dexamethasone sodium phosphate | 6030200 |
| 18572811000001100 | Methylprednisolone acetate | 06030200/10010202 |
| 3231811000001100 | Methylprednisolone acetate | 06030200/10010202 |
| 18573011000001100 | Methylprednisolone acetate/Lidocaine hydrochloride | 10010202 |
| 769111000001105 | Methylprednisolone acetate/Lidocaine hydrochloride | 10010202 |
| 17341411000001100 | Methylprednisolone acetate | 06030200/10010202 |
| 36034611000001100 | Methylprednisolone acetate | 06030200/10010202 |
| 470611000001109 | Methylprednisolone acetate/Lidocaine hydrochloride | 10010202 |
| 623611000001104 | Methylprednisolone acetate | 06030200/10010202 |
| 16157011000001100 | Methylprednisolone acetate | 06030200/10010202 |
| 355311000001102 | Prednisolone acetate | 08020200/10010201/10010202 |
| 16157411000001100 | Methylprednisolone acetate/Lidocaine hydrochloride | 10010202 |
| 17341211000001100 | Methylprednisolone acetate/Lidocaine hydrochloride | 10010202 |
| 11104811000001100 | Methylprednisolone acetate | 06030200/10010202 |
| 37420111000001100 | Methylprednisolone acetate/Lidocaine hydrochloride | 10010202 |
| 36034811000001100 | Methylprednisolone acetate | 06030200/10010202 |
| 777311000001106 | Methylprednisolone acetate | 06030200/10010202 |
| 36034411000001100 | Methylprednisolone acetate/Lidocaine hydrochloride | 10010202 |
| 36021411000001100 | Prednisolone acetate | 08020200/10010201/10010202 |
| 36034711000001100 | Methylprednisolone acetate | 06030200/10010202 |
| 36034511000001100 | Methylprednisolone acetate/Lidocaine hydrochloride | 10010202 |
| 325476008 | Deflazacort | 6030200 |
| 17917211000001100 | Prednisolone | 01050200/03020000/06030200/08020200/10010201 |
| 325474006 | Deflazacort | 0 |
| 707911000001100 | Prednisolone | 01050200/03020000/05011000/05040800/06030200/08020200/10010201 |
| 25615001000027100 | Prednisolone | 6030200 |
| 110811000001100 | Prednisolone | 01050200/03020000/05011000/05040800/06030200/08020200/10010201 |
| 4051211000001100 | Methylprednisolone | 6030200 |
| 3960211000001100 | Deflazacort | 0 |
| 3286911000001100 | Dexamethasone | 06030200/64090000 |
| 30114811000001100 | Prednisolone | 01050200/03020000/05011000/05040800/06030200/08020200/10010201 |
| 18285211000001100 | Prednisolone | 01050200/03020000/05011000/05040800/06030200/08020200/10010201 |
| 3292011000001100 | Dexamethasone | 06030200/64090000 |
| 28799311000001100 | Prednisolone | 01050200/03020000/05011000/05040800/06030200/08020200/10010201 |
| 224015001000027000 | Deflazacort | 06030200/08020200 |
| 325450008 | Prednisolone | 01050200/03020000/06030200/08020200/10010201 |
| 111515001000027000 | Prednisolone | 6030200 |
| 28799011000001100 | Prednisolone | 01050200/03020000/05011000/05040800/06030200/08020200/10010201 |
| 716111000001103 | Prednisolone | 01050200/03020000/06030200/08020200/10010201 |
| 15628711000001100 | Dexamethasone | 06030200/64090000 |
| 41195001000027100 | Dexamethasone | 6030200 |
| 325472005 | Deflazacort | 6030200 |
| 28808711000001100 | Prednisolone | 01050200/03020000/05011000/05040800/06030200/08020200/10010201 |
| 238511000001107 | Prednisolone | 01050200/03020000/05011000/05040800/06030200/08020200/10010201 |
| 18935001000027100 | Prednisolone | 6030200 |
| 844111000001103 | Prednisolone | 01050200/03020000/05011000/05040800/06030200/08020200/10010201 |
| 325426006 | Prednisolone | 01050200/03020000/05011000/05040800/06030200/08020200/10010201 |
| 28475001000027100 | Prednisolone Steaglate | 01050200/06030200 |
| 325412002 | Methylprednisolone | 6030200 |
| 649711000001109 | Prednisolone | 01050200/03020000/05011000/05040800/06030200/08020200/10010201 |
| 4852811000001100 | Prednisolone | 01050200/03020000/06030200/08020200/10010201 |
| 350611000001104 | Prednisolone | 01050200/03020000/05011000/05040800/06030200/08020200/10010201 |
| 28799611000001100 | Prednisolone | 01050200/03020000/05011000/05040800/06030200/08020200/10010201 |
| 79935001000027100 | Prednisolone | 6030200 |
| 29805001000027100 | Prednisolone | 6030200 |
| 47615001000027100 | Prednisolone | 6030200 |
| 29825001000027100 | Prednisone | 01050200/06030200 |
| 21851411000001100 | Prednisolone | 01050200/03020000/05011000/05040800/06030200/08020200/10010201 |
| 325356003 | Dexamethasone | 06030200/64090000 |
| 17967911000001100 | Dexamethasone | 06030200/64090000 |
| 18945001000027100 | Prednisolone | 6030200 |
| 37743911000001100 | Dexamethasone | 6030200 |
| 52911000001107 | Prednisolone | 01050200/03020000/05011000/05040800/06030200/08020200/10010201 |
| 3804311000001100 | Betamethasone | 6030200 |
| 34796811000001100 | Dexamethasone | 8020200 |
| 876911000001107 | Prednisolone | 01050200/03020000/05011000/05040800/06030200/08020200/10010201 |
| 3291411000001100 | Dexamethasone | 06030200/64090000 |
| 325411009 | Methylprednisolone | 6030200 |
| 646211000001106 | Prednisolone | 01050200/03020000/05011000/05040800/06030200/08020200/10010201 |
| 325345003 | Betamethasone | 6030200 |
| 374072009 | Prednisone | 6030200 |
| 418349006 | Prednisone | 6030200 |
| 325413007 | Methylprednisolone | 6030200 |
| 35574111000001100 | Dexamethasone | 6030200 |
| 18285011000001100 | Prednisolone | 01050200/03020000/05011000/05040800/06030200/08020200/10010201 |
| 9807711000001100 | Prednisolone | 01050200/03020000/05011000/05040800/06030200/08020200/10010201 |
| 4052211000001100 | Methylprednisolone | 6030200 |
| 32774411000001100 | Prednisolone | 01050200/03020000/05011000/05040800/06030200/08020200/10010201 |
| 325445002 | Prednisolone | 0 |
| 325427002 | Prednisolone | 01050200/03020000/05011000/05040800/06030200/08020200/10010201 |
| 21851211000001100 | Prednisolone | 01050200/03020000/05011000/05040800/06030200/08020200/10010201 |
| 14786411000001100 | Prednisolone | 01050200/03020000/05011000/05040800/06030200/08020200/10010201 |
| 111525001000027000 | Prednisolone | 6030200 |
| 6935001000027100 | Prednisone | 01050200/06030200 |
| 30991311000001100 | Prednisolone | 01050200/03020000/06030200/08020200/10010201 |
| 28808611000001100 | Prednisolone | 01050200/03020000/05011000/05040800/06030200/08020200/10010201 |
| 940311000001100 | Prednisolone | 01050200/03020000/05011000/05040800/06030200/08020200/10010201 |
| 325410005 | Methylprednisolone | 6030200 |
| 32781411000001100 | Prednisolone | 01050200/03020000/05011000/05040800/06030200/08020200/10010201 |
| 9807911000001100 | Prednisolone | 01050200/03020000/05011000/05040800/06030200/08020200/10010201 |
| 25625001000027100 | Prednisolone | 6030200 |
| 28799811000001100 | Prednisolone | 01050200/03020000/06030200/08020200/10010201 |
| 325355004 | Dexamethasone | 06030200/64090000 |
| 3850711000001100 | Deflazacort | 6030200 |
| 4051911000001100 | Methylprednisolone | 6030200 |
| 3756411000001100 | Deflazacort | 6030200 |
| 31195001000027100 | Prednisone | 01050200/06030200 |
| 191525001000027000 | Dexamethasone | 6030200 |
| 3286511000001100 | Dexamethasone | 06030200/64090000 |
| 14786811000001100 | Prednisolone | 01050200/03020000/05011000/05040800/06030200/08020200/10010201 |
| 28808411000001100 | Prednisolone | 01050200/03020000/05011000/05040800/06030200/08020200/10010201 |
| 3290311000001100 | Dexamethasone | 06030200/64090000 |
| 85511000001101 | Prednisolone | 01050200/03020000/05011000/05040800/06030200/08020200/10010201 |
| 373994007 | Prednisone | 6030200 |
| 32808311000001100 | Prednisolone | 01050200/03020000/05011000/05040800/06030200/08020200/10010201 |
| 376688006 | Dexamethasone | 6030200 |
| 28798211000001100 | Prednisolone | 01050200/03020000/05011000/05040800/06030200/08020200/10010201 |
| 65425001000027100 | Prednisolone | 6030200 |
| 18625211000001100 | Prednisolone | 01050200/03020000/05011000/05040800/06030200/08020200/10010201 |
| 4051611000001100 | Methylprednisolone | 6030200 |
| 3287811000001100 | Dexamethasone | 06030200/64090000 |
| 47605001000027100 | Prednisolone | 6030200 |
| 244685001000027000 | Prednisolone | 6030200 |
| 137755001000027000 | Prednisolone | 6030200 |
| 137775001000027000 | Prednisolone Steaglate | 01050200/06030200 |

**Myocardial infarction codes**

| **Medcode** | **Read term** |
| --- | --- |
| 241 | Acute myocardial infarction |
| 1204 | Heart attack |
| 1677 | MI - acute myocardial infarction |
| 1678 | Inferior myocardial infarction NOS |
| 2491 | Coronary thrombosis |
| 3704 | Acute subendocardial infarction |
| 5387 | Other specified anterior myocardial infarction |
| 8935 | Acute inferolateral infarction |
| 9276 | Acute coronary insufficiency |
| 9507 | Acute non-Q wave infarction |
| 10562 | Acute non-ST segment elevation myocardial infarction |
| 11983 | Acute coronary syndrome |
| 12139 | Acute anterolateral infarction |
| 12229 | Acute ST segment elevation myocardial infarction |
| 13566 | Attack - heart |
| 13571 | Thrombosis - coronary |
| 14658 | Acute myocardial infarction NOS |
| 14897 | Anterior myocardial infarction NOS |
| 14898 | Lateral myocardial infarction NOS |
| 17689 | Silent myocardial infarction |
| 17872 | Acute anteroseptal infarction |
| 18842 | Subsequent myocardial infarction |
| 23708 | Atrial septal defect/curr comp folow acut myocardal infarct |
| 23892 | Posterior myocardial infarction NOS |
| 24126 | Haemopericardium/current comp folow acut myocard infarct |
| 27951 | Other acute and subacute ischaemic heart disease |
| 27977 | Other acute and subacute ischaemic heart disease NOS |
| 28736 | Acute atrial infarction |
| 29421 | Silent myocardial ischaemia |
| 29553 | Thrombosis atrium,auric append&vent/curr comp foll acute MI |
| 29643 | Acute inferoposterior infarction |
| 29758 | Acute transmural myocardial infarction of unspecif site |
| 30330 | Acute Q-wave infarct |
| 30421 | Cardiac rupture following myocardial infarction (MI) |
| 32272 | Postoperative myocardial infarction |
| 32854 | Acute posterolateral myocardial infarction |
| 34803 | Other acute myocardial infarction |
| 36423 | Certain current complication follow acute myocardial infarct |
| 36523 | Preinfarction syndrome |
| 37657 | Ventric septal defect/curr comp fol acut myocardal infarctn |
| 38609 | Subsequent myocardial infarction of inferior wall |
| 39693 | Subendocardial ischaemia |
| 40429 | Acute anteroapical infarction |
| 41221 | Acute septal infarction |
| 41835 | Postoperative subendocardial myocardial infarction |
| 45809 | Subsequent myocardial infarction of anterior wall |
| 46017 | Other acute myocardial infarction NOS |
| 46112 | Postoperative transmural myocardial infarction anterior wall |
| 46166 | Subsequent myocardial infarction of unspecified site |
| 46276 | Postoperative transmural myocardial infarction inferior wall |
| 54251 | Preinfarction syndrome NOS |
| 59189 | Ruptur cardiac wall w'out haemopericard/cur comp fol ac MI |
| 59940 | Ruptur chordae tendinae/curr comp fol acute myocard infarct |
| 61670 | Diab mellit insulin-glucose infus acute myocardial infarct |
| 62626 | Acute papillary muscle infarction |
| 63467 | True posterior myocardial infarction |
| 68357 | Microinfarction of heart |
| 68748 | Postoperative myocardial infarction, unspecified |
| 69474 | Rupture papillary muscle/curr comp fol acute myocard infarct |
| 72562 | Subsequent myocardial infarction of other sites |
| 95550 | Admit ischaemic heart disease emergency |
| 96838 | [X]Acute transmural myocardial infarction of unspecif site |
| 99991 | [X]Subsequent myocardial infarction of unspecified site |
| 106812 | Postoperative transmural myocardial infarction unspec site |
| 109035 | [X]Subsequent myocardial infarction of other sites |
| 35119 | Post infarction pericarditis |
| **ICD-10** | **Term** |
| I21 (0,1,2,3,4,9) | Acute myocardial infarction |
| I22 (0,1,2,8,9) | Subsequent myocardial infarction |
| I23 (0,1,2,3,4,5,6,8) | Certain current complications following acute myocardial infarction |
| **ICD-9** | **Term** |
| 410 | Acute myocardial infarction |
| 411 | Postmyocardial infarction syndrome |

**Acute cerebrovascular accident codes**

| **Medcode** | **Read term** |
| --- | --- |
| 504 | Transient cerebral ischaemia |
| 569 | Infarction - cerebral |
| 1298 | CVA unspecified |
| 1433 | Transient ischaemic attack |
| 1469 | Stroke and cerebrovascular accident unspecified |
| 1786 | Subarachnoid haemorrhage |
| 1895 | Transient cerebral ischaemia NOS |
| 3149 | Cerebral infarction NOS |
| 3535 | Intracerebral haemorrhage NOS |
| 4152 | Thrombosis, carotid artery |
| 4240 | Carotid artery occlusion |
| 5051 | Intracerebral haemorrhage |
| 5185 | Lateral medullary syndrome |
| 5363 | CVA - cerebral artery occlusion |
| 5602 | Cerebellar infarction |
| 6116 | CVA - Cerebrovascular accident unspecified |
| 6155 | Stroke due to cerebral arterial occlusion |
| 6253 | Stroke unspecified |
| 6960 | CVA - cerebrovascular accid due to intracerebral haemorrhage |
| 7780 | Left sided CVA |
| 7912 | Pontine haemorrhage |
| 8443 | Brain stem stroke syndrome |
| 8837 | erebral arterial occlusion |
| 9696 | Subarachnoid haemorrhage from posterior communicating artery |
| 9985 | Left sided cerebral infarction |
| 10504 | Right sided cerebral infarction |
| 12833 | Right sided CVA |
| 13564 | Cerebellar haemorrhage |
| 15019 | Cerebral embolism |
| 15252 | Brainstem infarction NOS |
| 15788 | Transient cerebral ischaemia NOS |
| 16507 | Intermittent cerebral ischaemia |
| 16517 | Cerebral thrombosis |
| 16956 | Cerebral palsy, not congenital or infantile, acute |
| 17322 | Cerebellar haemorrhage |
| 17326 | Cerebral embolism |
| 18604 | Cerebral thrombosis |
| 18689 | Cerebrl infarctn due/unspcf occlusn or sten/cerebrl artrs |
| 19201 | Cerebral embolus |
| 19260 | Cerebral infarction due to thrombosis of cerebral arteries |
| 19280 | Cereb infarct due cerebral venous thrombosis, nonpyogenic |
| 19354 | Other transient cerebral ischaemia |
| 19412 | Subarachnoid haemorrhage from middle cerebral artery |
| 20284 | Intracranial haemorrhage NOS |
| 23580 | Subarachnoid haemorrhage NOS |
| 23671 | Cerebral infarct due to thrombosis of precerebral arteries |
| 24446 | Cerebral infarction due to embolism of precerebral arteries |
| 25615 | Brainstem infarction |
| 26424 | Infarction of basal ganglia |
| 27975 | Cerebral infarction due to embolism of cerebral arteries |
| 28314 | Left sided intracerebral haemorrhage, unspecified |
| 29939 | Ruptured berry aneurysm |
| 30045 | External capsule haemorrhage |
| 30202 | Intracerebral haemorrhage, intraventricular |
| 31060 | Intracerebral haemorrhage in hemisphere, unspecified |
| 31595 | Cortical haemorrhage |
| 31704 | Occlusion/stenosis cerebral arts not result cerebral infarct |
| 31805 | Other and unspecified intracranial haemorrhage |
| 33499 | Pure motor lacunar syndrome |
| 33543 | Cerebrl infarctn due/unspcf occlusn or sten/cerebrl artrs |
| 34758 | Cerebral embolus |
| 36717 | Cerebral infarction due to thrombosis of cerebral arteries |
| 39344 | Cereb infarct due cerebral venous thrombosis, nonpyogenic |
| 40338 | Internal capsule haemorrhage |
| 40758 | Cereb infarct due unsp occlus/stenos precerebr arteries |
| 41910 | Subarachnoid haemorrhage from basilar artery |
| 42331 | Subarachnoid haemorrhage from anterior communicating artery |
| 44765 | Carotid artery syndrome hemispheric |
| 46316 | Basal nucleus haemorrhage |
| 47642 | Wallenberg syndrome |
| 50594 | Multiple and bilateral precerebral artery syndromes |
| 51759 | Occlusion and stenosis of middle cerebral artery |
| 51767 | Pure sensory lacunar syndrome |
| 53745 | [X]Other cerebral infarction |
| 53755 | [X]Cerebral palsy and other paralytic syndromes |
| 53810 | [X]Other intracerebral haemorrhage |
| 55602 | Occlusion and stenosis of cerebellar arteries |
| 56007 | Subarachnoid haemorrhage from carotid siphon and bifurcation |
| 57315 | Intracerebral haemorrhage, multiple localized |
| 57495 | Infarction - precerebral |
| 57527 | Occlusion and stenosis of anterior cerebral artery |
| 60692 | Subarachnoid haemorrhage from vertebral artery |
| 62342 | Bulbar haemorrhage |
| 63746 | [X]Other transnt cerebral ischaemic attacks+related syndroms |
| 65745 | [X]Other subarachnoid haemorrhage |
| 65770 | Occlusion and stenosis of posterior cerebral artery |
| 71274 | Occlusion+stenosis of multiple and bilat cerebral arteries |
| 71585 | Precerebral artery occlusion NOS |
| 90572 | [X]Occlusion and stenosis of other precerebral arteries |
| 91627 | [X]Cerebrl infarctn due/unspcf occlusn or sten/cerebrl artrs |
| 92036 | [X]Occlusion and stenosis of other cerebral arteries |
| 94482 | [X]Cereb infarct due unsp occlus/stenos precerebr arteries |
| 96630 | [X]Intracerebral haemorrhage in hemisphere, unspecified |
| 98642 | Multiple and bilateral precerebral arterial occlusion |
| 101733 | Cerebral vein thrombosis |
| 105738 | Carotid territory transient ischaemic attack |
| 107440 | Lobar cerebral haemorrhage |
| 108630 | [X]Subarachnoid haemorrh from intracranial artery, unspecif |
| 108668 | [X]Subarachnoid haemorrhage from other intracranial arteries |
| 55247 | Impending cerebral ischaemia |
| 2418 | Cerebrovascular disease |
| **ICD-10** | **Term** |
| I60 (0,1,2,3,4,5,6,7,8,9) | Subarachnoid haemorrhage |
| I61 (0,1,2,3,4,5,6,8,9) | Intracerebral haemorrhage |
| I63 (0,1,2,3,4,5,6,8,9) | Cerebral infarction |
| I64 | Stroke, not specified as haemorrhage or infarction |
| G45 (0,1,2,3,4,8,9) | Transient cerebral ischaemic attacks and related syndromes |
| **ICD-9** | **Term** |
| 430 | Subarachnoid haemorrhage |
| 431 | Intracerebral haemorrhage |
| 433 | Occlusion and stenosis of precerebral arteries |
| 434 | Occlusion of cerebral arteries |
| 435 | Transient cerebral ischemia |
| 436 | Acute, but ill-defined, cerebrovascular disease |

**First-ever consultation for cataract codes**

| **Medcode** | **Read Term** |
| --- | --- |
| 63640 | Morgagni cataract |
| 58625 | Cataract associated with other syndromes |
| 70201 | [X]Other specified cataract |
| 11255 | Subcapsular cataract |
| 4358 | Other cataract |
| 44260 | Insulin dependent diabetes mellitus with diabetic cataract |
| 50932 | Coronary cataract |
| 57805 | Unspecified presenile cataract |
| 8130 | Posterior capsule opacification |
| 22022 | Cortical senile cataract |
| 18048 | Lens capsule pseudoexfoliation |
| 1622 | O/E - cataract present |
| 45190 | Lamellar zonular cataract |
| 44294 | Immature cortical cataract |
| 48192 | Type II diabetes mellitus with diabetic cataract |
| 18089 | Capsular cataract |
| 41668 | Infantile, juvenile and presenile cataracts |
| 49554 | Type 1 diabetes mellitus with diabetic cataract |
| 6330 | Extracapsular extraction of cataract |
| 44794 | Unspecified infantile cataract |
| 47566 | Unspecified senile cataract |
| 9931 | O/E - Left cataract present |
| 51162 | Hypermature cataract |
| 58078 | Nonsenile cataract NOS |
| 10659 | Diabetic cataract |
| 6876 | Nuclear senile cataract |
| 98962 | Cortical or zonular cataract NOS |
| 59125 | Anterior subcapsular polar cataract |
| 44982 | Type 2 diabetes mellitus with diabetic cataract |
| 11941 | Referral to cataract clinic |
| 92358 | Anterior subcapsular polar senile cataract |
| 90279 | Other congenital cataract or lens anomaly NOS |
| 54130 | Cataract in eye inflammatory disorder |
| 70700 | Other specified congenital cataract or lens anomaly |
| 4148 | Congenital cataract and lens anomalies |
| 88738 | Capsular or subcapsular cataract NOS |
| 6317 | Cataract NOS |
| 15589 | Other cataract NOS |
| 70403 | Other nonsenile cataract |
| 62188 | Unspecified cataracta complicata |
| 29770 | Senile cataract NOS |
| 49085 | Other senile cataract |
| 69278 | Non-insulin depend diabetes mellitus with diabetic cataract |
| 24467 | Unspecified secondary cataract |
| 96385 | Cortical and zonular cataract |
| 63491 | Unspecified juvenile cataract |
| 99424 | Congenital membranous cataract |
| 19371 | Cataract in degenerative disorder |
| 49297 | [X]Cataract in other diseases classified elsewhere |
| 70257 | Combined senile cataract |
| 703 | Bilateral cataracts |
| 93727 | Type II diabetes mellitus with diabetic cataract |
| 100770 | Insulin dependent diabetes mellitus with diabetic cataract |
| 5325 | Posterior subcapsular polar senile cataract |
| 26097 | Punctate cataract |
| 45926 | Congenital cataract or lens anomaly NOS |
| 94430 | Cataract due to other disorder NOS |
| 44779 | Type 2 diabetes mellitus with diabetic cataract |
| 23883 | Needling of lens for cataract |
| 71623 | Total or subtotal congenital cataract NOS |
| 94474 | Combined nonsenile cataract |
| 48228 | Cataract secondary to ocular disease |
| 6547 | O/E - Right cataract present |
| 101939 | [X]Other senile cataract |
| 23631 | Immature cataract NOS |
| 46169 | Cataract secondary to ocular disorder NOS |
| 48148 | Incipient cataract NOS |
| 59914 | Capsular and subcapsular cataract |
| 296 | Cataract |
| 110400 | Type 1 diabetes mellitus with diabetic cataract |
| 4260 | Intracapsular extraction of cataract |
| 63429 | Zonular cataract |
| 299 | Congenital cataract, unspecified |
| 45952 | Tetanic cataract |
| 7257 | Cortical cataract |
| 10010 | Senile cataract |
| 64196 | Cataract observation |
| 61325 | Cataract with neovascularization |
| 71291 | Cataract due to other disorder |
| 33793 | Total, mature senile cataract |
| 60883 | Myotonic cataract |
| 7793 | Nuclear cataract |
| 17545 | Type I diabetes mellitus with diabetic cataract |
| 11767 | Referral for cataract extraction |
| 27898 | Blue dot cataract |
| 42452 | Vitreous syndrome following cataract surgery |
| 4242 | Posterior subcapsular polar cataract |
| **ICD10 codes** | **Term** |
| H26.0 | Infantile, juvenile and presenile cataract |
| H28 | Cataract and other disorders of lens in diseases classified elsewhere |
| Q12.0 | Congenital cataract |
| H25 | Senile cataract |
| H26.2 | Complicated cataract |
| H26.9 | Cataract, unspecified |
| H26.8 | Other specified cataract |

**Supplementary Material** **S4**. Results of sensitivity analyses of the self-controlled case series study analyses of cardiovascular event outcomes for people diagnosed with gout for the first time according to the broad definition (definition 1).

**
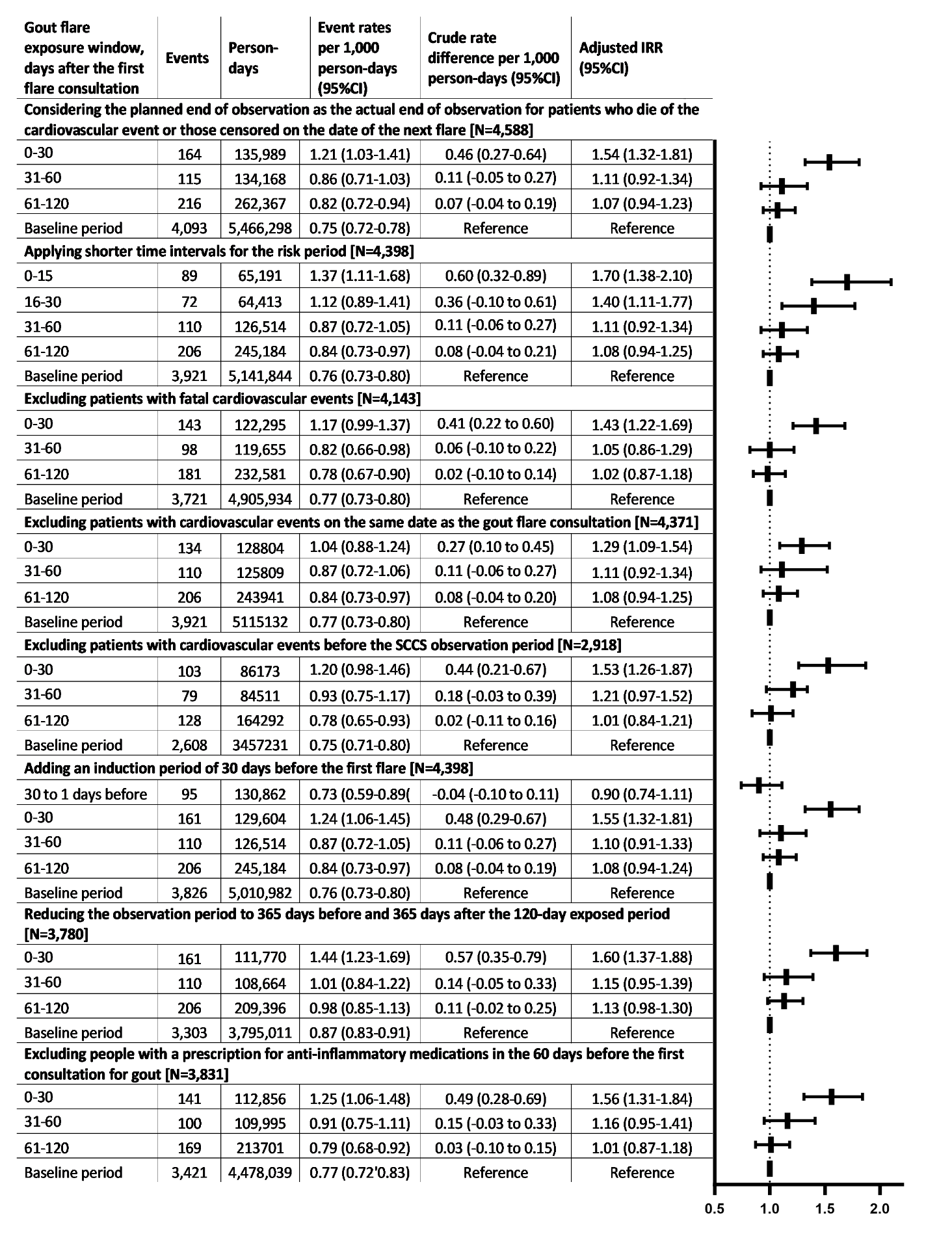
**

*The number of patients included in each analysis is reported in square brackets. The analyses were adjusted for age and calendar season. The baseline interval consisted of a pre-exposure period of up to 730 days before the first consultation for gout and a post-exposure period of up to 610 days after the exposed period at most. 95%CI: 95% confidence interval, aIRR: adjusted incidence rate ratio, SCCS: self-controlled case-series.*

| **Supplementary Material S5**. Results of stratified analyses of the self-controlled case series study analysis of cardiovascular event outcomes for people diagnosed with gout for the first time according to the broad definition (definition 1). | | | | | |
| --- | --- | --- | --- | --- | --- |
| **Gout flare exposure window, days after the first gout consultation** | **Events** | **Person-days** | **Event rates per 1,000 person-days (95%CI)** | **Crude rate difference per 1,000 person-days (95%CI)** | **Adjusted IRR (95%CI)** |
| **Age >70 years [N=3,043]** | | | | | |
| 0-30 | 122 | 89,604 | 1.36 (1.14-1.63) | 0.59 (0.35-0.84) | 1.68 (1.40-2.01) |
| 31-60 | 84 | 87,305 | 0.96 (0.77-1.20) | 0.19 (-0.15 to 0.40) | 1.21 (0.97-1.51) |
| 61-120 | 149 | 168,259 | 0.89 (0.75-1.04) | 0.12 (-0.03 to 0.26) | 1.13 (0.96-1.33) |
| Baseline period | 2,688 | 3,492,998 | 0.77 (0.73-0.81) | Reference | Reference |
| **Age ≤70 years [N=1,355]** | | | | | |
| 0-30 | 39 | 40,000 | 0.98 (0.71-1.34) | 0.23 (-0.02 to 0.54) | 1.27 (0.96-1.74) |
| 31-60 | 26 | 39,209 | 0.66 (0.45-0.98) | -0.09 (-0.34 to 0.17) | 0.87 (0.59-1.29) |
| 61-120 | 57 | 76,925 | 0.74 (0.57-0.97) | 0.00 (-0.20 to 0.19) | 0.98 (0.75-1.28) |
| Baseline period | 1,233 | 1,648,846 | 0.75 (0.69-0.81) | Reference | Reference |
| **Males [N=2,942]** | | | | | |
| 0-30 | 93 | 86,610 | 1.07 (0.87-1.32) | 0.31 (0.09 to 0.53) | 1.35 (1.10-1.67) |
| 31-60 | 68 | 84,669 | 0.80 (0.63-1.02) | 0.04 (-0.15 to 0.23) | 1.03 (0.81-1.31) |
| 61-120 | 134 | 164,293 | 0.82 (0.69-0.97) | 0.05 (-0.09 to 0.19) | 1.05 (0.88-1.25) |
| Baseline period | 2,647 | 3,461,539 | 0.76 (0.72-0.81) | Reference | Reference |
| **Females [N=1,456]** | | | | | |
| 0-30 | 68 | 42,994 | 1.58 (1.24-2.02) | 0.82 (0.45-1.20) | 1.96 (1.53-2.51) |
| 31-60 | 42 | 41,845 | 1.00 (0.74-1.36) | 0.25 (-0.06 to 0.55) | 1.28 (0.94-1.74) |
| 61-120 | 72 | 80,891 | 0.89 (0.70-1.13) | 0.13 (-0.08 to 0.34) | 1.16 (0.91-1.48) |
| Baseline period | 1,274 | 1,680,305 | 0.76 (0.70-0.82) | Reference | Reference |
| **Patients with serum urate levels >480 micromol/l [N=1,731]** | | | | | |
| 0-30 | 78 | 50,354 | 1.55 (1.23-1.95) | 0.75 (0.40 to 1.10) | 1.81 (1.44-2.27) |
| 31-60 | 47 | 48,684 | 0.97 (0.73-1.3) | 0.17 (-0.11 to 0.45) | 1.16 (0.87-1.55) |
| 61-120 | 87 | 92,978 | 0.94 (0.76-1.17) | 0.14 (-0.06 to 0.34) | 1.15 (0.92-1.43) |
| Baseline period | 1,519 | 1,900,516 | 0.80 (0.75-0.86) | Reference | Reference |
| **Patients with arterial hypertension $ [N=2,896]** | | | | | |
| 0-30 | 100 | 86,087 | 1.16 (0.95 to 1.42) | 0.40 (0.17 to 0.63) | 1.45 (1.19-1.77) |
| 31-60 | 66 | 84,000 | 0.79 (0.62 to 1.01) | 0.03 (-0.16 to 0.22) | 1.01 (0.79-1.28) |
| 61-120 | 143 | 162,381 | 0.88 (0.74 to 1.04) | 0.12 (-0.03 to 0.27) | 1.15 (0.97-1.36) |
| Baseline period | 2,587 | 3,409,290 | 0.76 (0.72 to 0.8) | Reference | Reference |
| **Patients without arterial hypertension $ [N=1,502]** | | | | | |
| 0-30 | 61 | 43,517 | 1.40 (1.08 to 1.81) | 0.63 (0.28 to 0.98) | 1.32 (1.01-1.79) |
| 31-60 | 44 | 42,514 | 1.03 (0.76 to 1.39) | 0.26 (-0.05 to 0.57) | 0.97 (0.75-1.25) |
| 61-120 | 63 | 82,803 | 0.76 (0.59 to 0.98) | -0.01 (-0.2 to 0.18) | 0.85 (0.65-1.12) |
| Baseline period | 1,334 | 1,732,554 | 0.77 (0.71 to 0.83) | Reference | Reference |
| **Patients with chronic kidney disease stage III-V $ [N=1,228]** | | | | | |
| 0-30 | 41 | 36,330 | 1.13 (0.83 to 1.54) | 0.34 (0.00 to 0.69) | 1.35 (1.00-1.83) |
| 31-60 | 25 | 35,071 | 0.71 (0.48 to 1.06) | -0.08 (-0.36 to 0.2) | 0.86 (0.58-1.28) |
| 61-120 | 66 | 66,793 | 0.99 (0.77 to 1.27) | 0.2 (-0.04 to 0.44) | 1.21 (0.94-1.55) |
| Baseline period | 1,096 | 1,392,498 | 0.79 (0.73 to 0.86) | Reference | Reference |
| **Patients without chronic kidney disease stage III-V $ [N=3,170]** | | | | | |
| 0-30 | 120 | 93,274 | 1.29 (1.07 to 1.55) | 0.54 (0.31 to 0.77) | 1.62 (1.35-1.94) |
| 31-60 | 85 | 91,443 | 0.93 (0.75 to 1.15) | 0.18 (-0.02 to 0.38) | 1.19 (0.96-1.48) |
| 61-120 | 140 | 178,391 | 0.78 (0.66 to 0.92) | 0.03 (-0.1 to 0.16) | 1.02 (0.86-1.21) |
| Baseline period | 2,825 | 3,749,346 | 0.75 (0.71 to 0.79) | Reference | Reference |
| **Patients with heart failure $ [N=981]** | | | | | |
| 0-30 | 39 | 28,837 | 1.35 (0.98 to 1.86) | 0.54 (0.11 to 0.97) | 1.57 (1.14-2.17) |
| 31-60 | 23 | 27,728 | 0.83 (0.55 to 1.26) | 0.02 (-0.32 to 0.36) | 0.98 (0.65-1.49) |
| 61-120 | 50 | 52,305 | 0.96 (0.72 to 1.28) | 0.15 (-0.12 to 0.42) | 1.15 (0.86-1.54) |
| Baseline period | 869 | 1,074,586 | 0.81 (0.74 to 0.89) | Reference | Reference |
| **Patients without heart failure $ [N=3,417]** | | | | | |
| 0-30 | 122 | 100,767 | 1.21 (1.01 to 1.45) | 0.46 (0.24 to 0.68) | 1.55 (1.29-1.85) |
| 31-60 | 87 | 98,786 | 0.88 (0.71 to 1.09) | 0.13 (-0.06 to 0.32) | 1.15 (0.93-1.42) |
| 61-120 | 156 | 192,879 | 0.81 (0.69 to 0.95) | 0.06 (-0.07 to 0.19) | 1.06 (0.9-1.25) |
| Baseline period | 3,052 | 4,067,258 | 0.75 (0.71 to 0.79) | Reference | Reference |
| **Patients with diabetes mellitus $ [N=930]** | | | | | |
| 0-30 | 39 | 27,626 | 1.41 (1.02 to 1.94) | 0.64 (0.19 to 1.09) | 1.71 (1.24-2.37) |
| 31-60 | 20 | 26,776 | 0.75 (0.48 to 1.17) | -0.02 (-0.35 to 0.31) | 0.93 (0.59-1.45) |
| 61-120 | 46 | 51,465 | 0.89 (0.66 to 1.2) | 0.12 (-0.14 to 0.38) | 1.14 (0.84-1.54) |
| Baseline period | 825 | 1,067,579 | 0.77 (0.7 to 0.85) | Reference | Reference |
| **Patients without diabetes mellitus $ [N=930]** | | | | | |
| 0-30 | 122 | 101,978 | 1.20 (1 to 1.44) | 0.44 (0.23 to 0.65) | 1.51 (1.26-1.81) |
| 31-60 | 90 | 99,738 | 0.90 (0.73 to 1.11) | 0.14 (-0.05 to 0.33) | 1.16 (0.94-1.43) |
| 61-120 | 160 | 193,719 | 0.83 (0.71 to 0.97) | 0.07 (-0.06 to 0.2) | 1.06 (0.9-1.25) |
| Baseline period | 3,096 | 4,074,265 | 0.76 (0.72 to 0.8) | Reference | Reference |
| **Patients with a number of diuretic prescriptions within the SCCS study period below the median value * [N2,184]** | | | | | |
| 0-30 | 82 | 63,898 | 1.28 (1.03 to 1.6) | 0.51 (0.23 to 0.79) | 1.57 (1.26-1.97) |
| 31-60 | 52 | 62,120 | 0.84 (0.64 to 1.11) | 0.07 (-0.16 to 0.3) | 1.06 (0.8-1.39) |
| 61-120 | 100 | 120,340 | 0.83 (0.68 to 1.01) | 0.06 (-0.11 to 0.23) | 1.07 (0.87-1.3) |
| Baseline period | 1,950 | 2,542,318 | 0.77 (0.72 to 0.82) | Reference | Reference |
| **Patients with a number of diuretic prescriptions within the SCCS study period above the median value * [N=2,214]** | | | | | |
| 0-30 | 79 | 65,706 | 1.20 (0.96 to 1.5) | 0.44 (0.17 to 0.71) | 1.53 (1.22-1.92) |
| 31-60 | 58 | 64,394 | 0.90 (0.69 to 1.17) | 0.14 (-0.09 to 0.37) | 1.16 (0.89-1.51) |
| 61-120 | 106 | 124,844 | 0.85 (0.7 to 1.03) | 0.09 (-0.07 to 0.25) | 1.10 (0.91-1.34) |
| Baseline period | 1,971 | 2,599,526 | 0.76 (0.71 to 0.81) | Reference | Reference |
| *The number of patients included in each analysis is reported in square brackets. The analyses were adjusted for age and calendar season. The baseline interval consisted of a pre-exposure period of up to 730 days before the first consultation for gout and a post-exposure period of up to 610 days after the exposed period at most. -/-: not reported due to Clinical Practice Research Datalink policy of not disclosing data for ≤5 patients. 95%CI: 95% confidence interval, aIRR: adjusted incidence rate ratio, SCCS: self-controlled case-series.*  ** The number of diuretic prescriptions over the SCCS study period was 16 (interquartile range 0-30).*  *$ Covariates were ascertained on the exposure date (i.e., the date of the first gout flare consultation).* | | | | | |

**Supplementary Material** **S6**. Results of sensitivity analyses of the self-controlled case series study analyses of cardiovascular event outcomes for people diagnosed with gout for the first time according to the strict definition (definition 2).

*
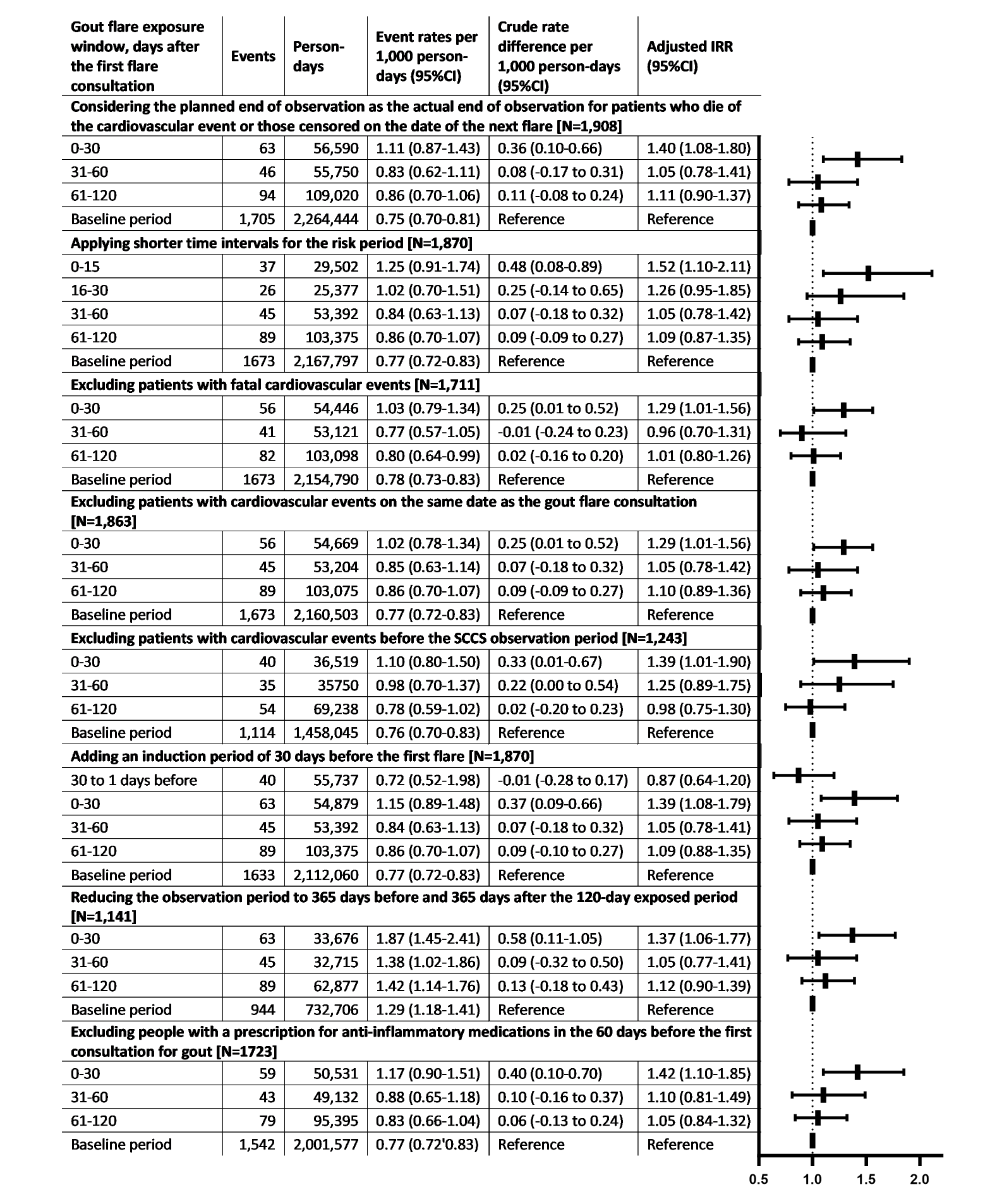
*

*The number of patients included in each analysis is reported in square brackets. The analyses were adjusted for age and calendar season. The baseline interval consisted of a pre-exposure period of up to 730 days before the first consultation for gout and a post-exposure period of up to 610 days after the exposed period at most. 95%CI: 95% confidence interval, aIRR: adjusted incidence rate ratio, SCCS: self-controlled case-series.*

| **Supplementary Material S7**. Results of stratified analyses of the self-controlled case series study analysis of cardiovascular event outcomes for people diagnosed with gout for the first time according to the strict definition (definition 2). | | | | | | |
| --- | --- | --- | --- | --- | --- | --- |
| **Gout flare exposure window, days after the first gout consultation** | **Events** | **Person-days** | **Event rates per 1,000 person-days (95%CI)** | **Crude rate difference per 1,000 person-days (95%CI)** | **Adjusted IRR (95%CI)** |  |
| **Age >70 years [N=1,319]** | | | | | | |
| 0-30 | 49 | 36,275 | 1.35 (1.01-1.83) | 0.53 (0.15-0.91) | 1.52 (1.14-2.03) |  |
| 31-60 | 35 | 35,212 | 0.99 (0.71-1.39 | 0.17 (-0.16 to 0.50) | 1.14 (0.81-1.60) |  |
| 61-120 | 70 | 67,881 | 1.03 (0.94-1.25) | 0.21 (-0.04 to 0.45) | 1.18 (0.92-1.52) |  |
| Baseline period | 1,165 | 1,413,614 | 0.82 (0.76-0.89) | Reference | Reference |  |
| **Age ≤70 years [N=551]** | | | | | | |
| 0-30 | 14 | 16,264 | 0.86 (0.51-1.46) | 0.02 (-0.39 to 0.44) | 1.10 (0.64-1.85) |  |
| 31-60 | 10 | 15,850 | 0.63 (0.34-1.18) | -0.07 (-0.50 to 0.30) | 0.81 (0.43-1.52) |  |
| 61-120 | 19 | 30,999 | 0.61 (0.39-0.97) | -0.15 (-0.42 to 0.10) | 0.78 (0.49-1.24) |  |
| Baseline period | 508 | 665,502 | 0.76 (0.68-0.86) | Reference | Reference |  |
| **Males [N=1,264]** | | | | | | |
| 0-30 | 36 | 36,830 | 0.98 (0.70-1.36) | 0.20 (-0.14 to 0.46) | 1.21 (0.87-1.69) |  |
| 31-60 | 24 | 35,972 | 0.67 (0.45-1.00) | -0.09 (-0.36 to 0.21) | 0.84 (0.56-1.26) |  |
| 61-120 | 55 | 69,857 | 0.79 (0.60-1.03) | 0.01 (-0.21 to 0.22) | 0.99 (0.75-1.31) |  |
| Baseline period | 1,145 | 1,468,900 | 0.78 (0.72-0.84) | Reference | Reference |  |
| **Females [N=606]** | | | | | | |
| 0-30 | 27 | 17,809 | 1.52 (1.03-2.23) | 0.76 (0.17 to 1.32) | 1.79 (1.20-2.65) |  |
| 31-60 | 21 | 17,190 | 1.22 (0.79-1.89) | 0.46 (-0.03 to 1.08) | 1.48 (0.95-2.30) |  |
| 61-120 | 34 | 33,098 | 1.03 (0.73-1.45) | 0.27 (-0.08 to 0.62) | 1.28 (0.89-1.83) |  |
| Baseline period | 524 | 689,814 | 0.76 (0.67-0.85) | Reference | Reference |  |
| **Colchicine-treated flares [N=914] £** | | | | | | |
| 0-30 | 19 | 26,878 | 0.71 (0.45-1.11) | -0.09 (-0.42 to 0.23) | 0.84 (0.53-1.33) |  |
| 31-60 | 16 | 26,160 | 0.61 (0.37-1.00) | 0.19 (0.49 to 0.12) | 0.74 (0.45-1.21) |  |
| 61-120 | 38 | 50,689 | 0.75 (0.54-1.04) | -0.05 (-0.29 to 0.19) | 0.89 (0.64-1.24) |  |
| Baseline period | 841 | 1,051,172 | 0.80 (0.83-0.88) | Reference | Reference |  |
| **Corticosteroid-treated flares [N=136] £** | | | | | | |
| 0-30 | 5 | 3,995 | 1.25 (0.51-3.06) | 0.46 (-0.65 to 1.57) | 1.50 (0.61-3.69) |  |
| 31-60 | -/- | 3,779 | 0.53 (0.13-2.14) | -0.26 (-1.01 to 0.49) | 0.62 (0.15-2.52) |  |
| 61-120 | 8 | 7,263 | 1.10 (0.54-2.25) | 0.31 (-0.47 to 1.09 | 1.31 (0.62-2.78) |  |
| Baseline period | 121 | 152,978 | 0.79 (0.61-1.02) | Reference | Reference |  |
| **Non-steroidal anti-inflammatory drug-treated flares [N=797] £** | | | | | | |
| 0-30 | 26 | 23,633 | 1.10 (0.74-1.63) | 0.36 (-0.07 to 0.79) | 1.39 (0.93-2.06) |  |
| 31-60 | 26 | 23,220 | 1.12 (0.76-1.66) | 0.38 (-0.05 to 0.81) | 1.44 (0.97-2.14) |  |
| 61-120 | 40 | 45,178 | 0.89 (0.64-1.22) | 0.15 (-0.13 to 0.43) | 1.16 (0.84-1.60) |  |
| Baseline period | 705 | 953,973 | 0.74 (0.67-0.82) | Reference | Reference |  |
| **Flares requiring hospitalisation [N=146]** | | | | | | |
| 0-30 | 13 | 4,353 | 2.99 (1.69-5.29) | 2.24 (0.61 to 3.87) | 3.78 (2.12-6.73) |  |
| 31-60 | -/- | 4,117 | 0.97 (0.36-2.63) | 0.23 (-0.74 to 1.19) | 1.23 (0.45-3.35) |  |
| 61-120 | 7 | 7,462 | 0.94 (0.44-2.01) | 0.19 (-0.52 to 0.90) | 1.22 (0.56-2.66) |  |
| Baseline period | 122 | 163,608 | 0.75 (0.58-0.96) | Reference | Reference |  |
| **Patients with serum urate levels >480 micromol/l [N=728]** | | | | | |  |
| 0-30 | 29 | 21,172 | 1.37 (0.94-1.99) | 0.57 (0.07 to 1.07) | 1.66 (1.14-2.44) |  |
| 31-60 | 25 | 20,380 | 1.23 (0.82-1.83) | 0.43 (-0.05 to 0.91) | 1.41 (0.99-2.01) |  |
| 61-120 | 39 | 38,613 | 1.01 (0.73-1.4) | 0.21 (-0.11 to 0.53) | 1.26 (0.91-1.75) |  |
| Baseline period | 635 | 797,000 | 0.8 (0.72-0.89) | Reference | Reference |  |
| **Patients with arterial hypertension $ [N=1,163]** | | | | | | |
| 0-30 | 39 | 34,273 | 1.14 (0.83-1.57) | 0.38 (0.02 to 0.74) | 1.39 (1.01-1.93) |  |
| 31-60 | 29 | 33,369 | 0.87 (0.6-1.26) | 0.11 (-0.21 to 0.43) | 1.10 (0.76-1.6) |  |
| 61-120 | 63 | 64,418 | 0.98 (0.76-1.26) | 0.22 (-0.03 to 0.47) | 1.26 (0.97-1.63) |  |
| Baseline period | 1,032 | 1,354,099 | 0.76 (0.7-0.83) | Reference | Reference |  |
| **Patients without arterial hypertension $ [N=597]** | | | | | | |
| 0-30 | 18 | 17,335 | 1.04 (0.65-1.66) | 0.25 (-0.23 to 0.73) | 1.27 (0.79-2.03) |  |
| 31-60 | 16 | 16,851 | 0.95 (0.58-1.56) | 0.16 (-0.31 to 0.63) | 1.17 (0.71-1.93) |  |
| 61-120 | 21 | 32,864 | 0.64 (0.41-0.99) | -0.15 (-0.43 to 0.13) | 0.78 (0.5-1.22) |  |
| Baseline period | 542 | 688,389 | 0.79 (0.7-0.89) | Reference | Reference |  |
| **Patients with chronic kidney disease stage III-V $ [N=528]** | | | | | | |
| 0-30 | 14 | 15,494 | 0.90 (0.53-1.53) | 0.10 (-0.38 to 0.58) | 1.06 (0.62-1.81) |  |
| 31-60 | 12 | 14,864 | 0.81 (0.46-1.44) | 0.01 (-0.45 to 0.47) | 0.95 (0.53-1.69) |  |
| 61-120 | 32 | 28,340 | 1.13 (0.79-1.62) | 0.33 (-0.07 to 0.73) | 1.31 (0.91-1.9) |  |
| Baseline period | 470 | 589,991 | 0.80 (0.70-0.91) | Reference | Reference |  |
| **Patients without chronic kidney disease stage III-V $ [N=1,232]** | | | | | | |
| 0-30 | 43 | 36,114 | 1.19 (0.88-1.61) | 0.43 (0.07 to 0.79) | 1.48 (1.09-2.01) |  |
| 31-60 | 33 | 35,356 | 0.93 (0.66-1.31) | 0.17 (-0.15 to 0.49) | 1.20 (0.85-1.7) |  |
| 61-120 | 52 | 68,942 | 0.75 (0.57-0.99) | -0.01 (-0.22 to 0.2) | 0.98 (0.74-1.3) |  |
| Baseline period | 1,104 | 1,452,497 | 0.76 (0.70-0.83) | Reference | Reference |  |
| **Patients with heart failure $ [N=415]** | | | | | | |
| 0-30 | 17 | 12,025 | 1.41 (0.87-2.29) | 0.59 (-0.09 to 1.27) | 1.63 (1-2.66) |  |
| 31-60 | 7 | 11,551 | 0.61 (0.29-1.29) | -0.21 (-0.67 to 0.25) | 0.71 (0.33-1.5) |  |
| 61-120 | 19 | 21,731 | 0.87 (0.55-1.38) | 0.05 (-0.35 to 0.45) | 1.04 (0.65-1.66) |  |
| Baseline period | 372 | 453,165 | 0.82 (0.71-0.95) | Reference | Reference |  |
| **Patients without heart failure $ [N=1,345]** | | | | | | |
| 0-30 | 40 | 39,583 | 1.01 (0.74-1.38) | 0.25 (-0.07 to 0.57) | 1.27 (0.92-1.74) |  |
| 31-60 | 38 | 38,669 | 0.98 (0.71-1.35) | 0.22 (-0.1 to 0.54) | 1.26 (0.92-1.75) |  |
| 61-120 | 65 | 75,551 | 0.86 (0.67-1.1) | 0.1 (-0.11 to 0.31) | 1.12 (0.87-1.44) |  |
| Baseline period | 1,202 | 1,589,323 | 0.76 (0.70-0.82) | Reference | Reference |  |
| **Patients with diabetes mellitus $ [N=400]** | | | | | | |
| 0-30 | 18 | 11,786 | 1.53 (0.95-2.46) | 0.76 (0.05 to 1.47) | 1.82 (1.12-2.94) |  |
| 31-60 | 7 | 11,368 | 0.62 (0.29-1.31) | -0.15 (-0.61 to 0.31) | 0.76 (0.36-1.62) |  |
| 61-120 | 21 | 21,805 | 0.96 (0.62-1.49) | 0.19 (-0.23 to 0.61) | 1.22 (0.78-1.91) |  |
| Baseline period | 354 | 457,023 | 0.77 (0.66-0.89) | Reference | Reference |  |
| **Patients without diabetes mellitus $ [N=1,360]** | | | | | | |
| 0-30 | 39 | 39,822 | 0.98 (0.71-1.35) | 0.21 (-0.10 to 0.52) | 1.23 (0.89-1.71) |  |
| 31-60 | 38 | 38,852 | 0.98 (0.71-1.35) | 0.21 (-0.10 to 0.52) | 1.21 (0.88-1.66) |  |
| 61-120 | 63 | 75,477 | 0.83 (0.64-1.07) | 0.06 (-0.15 to 0.27) | 1.06 (0.82-1.37) |  |
| Baseline period | 1,220 | 1,585,465 | 0.77 (0.71-0.83) | Reference | Reference |  |
| **Patients with a number of diuretic prescriptions within the SCCS study period below the median value * [N=847]** | | | | | | |
| 0-30 | 27 | 24,733 | 1.09 (0.74-1.60) | 0.32 (-0.10 to 0.74) | 1.31 (0.89-1.93) |  |
| 31-60 | 22 | 24,025 | 0.92 (0.60-1.41) | 0.15 (-0.24 to 0.54) | 1.14 (0.74-1.74) |  |
| 61-120 | 39 | 46,486 | 0.84 (0.61-1.16) | 0.07 (-0.20 to 0.34) | 1.03 (0.74-1.43) |  |
| Baseline period | 759 | 984,460 | 0.77 (0.70-0.85) | Reference | Reference |  |
| **Patients without a number of diuretic prescriptions within the SCCS study period above the median value * [N=913]** | | | | | | |
| 0-30 | 30 | 26,875 | 1.12 (0.78-1.61) | 0.35 (-0.05 to 0.75) | 1.39 (0.97-2.01) |  |
| 31-60 | 23 | 26,195 | 0.88 (0.58-1.33) | 0.11 (-0.25 to 0.47) | 1.10 (0.73-1.67) |  |
| 61-120 | 45 | 50,796 | 0.89 (0.66-1.2) | 0.12 (-0.14 to 0.38) | 1.12 (0.82-1.52) |  |
| Baseline period | 815 | 1,058,028 | 0.77 (0.7-0.85) | Reference | Reference |  |
| *The number of patients included in each analysis is reported in square brackets. The analyses were adjusted for age and calendar season. The baseline interval consisted of a pre-exposure period of up to 730 days before the first consultation for gout and a post-exposure period of up to 610 days after the exposed period at most. -/-: not reported due to Clinical Practice Research Datalink policy of not disclosing data for ≤5 patients. 95%CI: 95% confidence interval, aIRR: adjusted incidence rate ratio, SCCS: self-controlled case-series.*  ** The number of diuretic prescriptions over the SCCS study period was 16 (interquartile range 0-30).*  *$ Covariates were ascertained on the exposure date (i.e., the date of the first gout flare consultation).* | | | | | | |

| **Supplementary Material S8.** Self-controlled case series analysis of negative control outcome for people diagnosed with gout for the first time according to the broad definition (definition 1). | | | | | |
| --- | --- | --- | --- | --- | --- |
| **Gout flare exposure window, days after the first gout consultation** | **Events** | **Person-days** | **Event rates per 1,000 person-days (95%CI)** | **Crude rate difference per 1,000 person-days (95%CI)** | **Adjusted IRR (95%CI)** |
| **Considering the first-ever consultation for cataract as the outcome [N=2,508]** | | | | | |
| 0-30 | 63 | 74,358 | 0.85 (0.66-1.09) | 0.10 (-0.11 to 0.32) | 1.11 (0.86-1.42) |
| 31-60 | 54 | 73,131 | 0.74 (0.56-0.97) | 0.00 (-0.20 to 0.20) | 0.98 (0.75-1.28) |
| 61-120 | 88 | 143,698 | 0.61 (0.49-0.76) | -0.13 (-0.26 to 0.01) | 0.83 (0.67-1.02) |
| Baseline period | 2,303 | 3,100,858 | 0.74 (0.70-0.79) | Reference | Reference |
| *The number of patients included in each analysis is reported in square brackets.*  *The analyses were adjusted for age and calendar season.*  *The baseline interval consisted of a pre-exposure period of up to 730 days before the first consultation for gout and a post-exposure period of up to 610 days after the exposed period at most.*  *95%CI: 95% confidence interval, aIRR: adjusted incidence rate ratio, SCCS: self-controlled case-series.* | | | | | |

| **Supplementary Material S9.** Self-controlled case series analysis of negative control outcome for people diagnosed with gout for the first time according to the strict definition for gout flare (definition 2). | | | | | |
| --- | --- | --- | --- | --- | --- |
| **Gout flare exposure window, days after the first gout consultation** | **Events** | **Person-days** | **Event rates per 1,000 person-days (95%CI)** | **Crude rate difference per 1,000 person-days (95%CI)** | **Adjusted IRR (95%CI)** |
| **Considering the first-ever consultation for cataract as the outcome [N=1,038]** | | | | | |
| 0-30 | 20 | 30,617 | 0.65 (0.42-1.02) | -0.10 (-0.39 to 0.19) | 0.85 (0.55-1.32) |
| 31-60 | 25 | 30,024 | 0.83 (0.56-1.24) | 0.08 (-0.25 to 0.41) | 1.10 (0.74-1.64) |
| 61-120 | 39 | 58,973 | 0.66 (0.48-0.91) | -0.09 (-0.30 to 0.13) | 0.89 (0.64-1.22) |
| Baseline period | 954 | 1,273,964 | 0.75 (0.68-0.82) | Reference | Reference |
| *The number of patients included in each analysis is reported in square brackets.*  *The analyses were adjusted for age and calendar season.*  *The baseline interval consisted of a pre-exposure period of up to 730 days before the first consultation for gout and a post-exposure period of up to 610 days after the exposed period at most.*  *95%CI: 95% confidence interval, aIRR: adjusted incidence rate ratio, SCCS: self-controlled case-series.* | | | | | |

| **Supplementary Material S10**. Incidence rate of first-ever consultation for cataract on days 1-30, 31-120, and 121-730 after first gout diagnosis. | | | | |
| --- | --- | --- | --- | --- |
| Days after the first gout flare consultation | Number of first-ever consultations for cataract | Person-time at risk (years) | Incidence rate per 1,000 patient-years at risk (95%CI) | Rate difference per 1,000 patient-years at risk (95%CI) |
| **Patients who met the inclusion criteria and fulfilled the broad definition for the first gout flare (definition-1) without a history of cataract** [N=69,875] | | | | |
| 1-30 | 57 | 5719.5 | 10.0 (7.7-12.9) | Reference |
| 31-120 | 160 | 16845.9 | 9.5 (8.1-11.1) | -0.5 (-3.4 to 2.5) |
| 121-730 | 1,102 | 101402.5 | 10.9 (10.2-11.5) | 0.9 (-1.8 to 3.6) |
| 1-730 | 1,319 | 123967.9 | 10.6 (10.1-11.2) | 0.7 (-2.0 to 3.3) |
| **Patients who met the inclusion criteria and fulfilled the strict definition for the first gout flare (definition-2)** **without a history of cataract** [N=24,225] | | | | |
| 1-30 | 23 | 1981.7 | 11.6 (7.7-17.5) | Reference |
| 31-120 | 72 | 5827.1 | 12.4 (9.8-15.6) | 0.8 (-4.8 to 6.3) |
| 121-730 | 425 | 34818.8 | 12.2 (11.1-13.4) | 0.6 (-4.3 to 5.5) |
| 1-730 | 520 | 42627.6 | 12.2 (11.2-13.3) | 0.6 (-4.2 to 5.4) |

**REFERENCES**

1 Cipolletta E, Tata LJ, Nakafero G, *et al.* Association between Gout Flare and Subsequent Cardiovascular Events among Patients with Gout. *JAMA*. 2022;328:440–50.

2 Clarson L, Hider S, Belcher J, *et al.* Increased risk of vascular disease associated with gout: a retrospective, matched cohort study in the UK clinical practice research datalink. *Ann Rheum Dis*. 2015;74:642–7.

3 Seminog O, Goldacre M. Gout as a risk factor for myocardial infarction and stroke in England: evidence from record linkage studies. *Rheumatology (Oxford)*. 2013;52:2251–9.

4 Whitaker HJ, Ghebremichael-Weldeselassie Y, Douglas IJ, *et al.* Investigating the assumptions of the self-controlled case series method. *Stat Med*. 2018;37:643–58.

5 Ghebremichael-Weldeselassie Y, Jabagi MJ, Botton J, *et al.* A modified self‐controlled case series method for event‐dependent exposures and high event‐related mortality, with application to COVID‐19 vaccine safety. *Stat Med*. 2022;41:1735.
